# B cell mechanobiology in health and disease: emerging techniques and insights into therapeutic responses

**DOI:** 10.1002/1873-3468.70071

**Published:** 2025-05-19

**Authors:** Marta Sampietro, Marco Cellani, Cristina Scielzo

**Affiliations:** ^1^ Unit of Malignant B Cells biology and 3D Modelling, Division of Experimental Oncology IRCCS Ospedale San Raffaele Milan Italy; ^2^ Università Vita‐Salute San Raffaele Milan Italy

**Keywords:** B cell, cytoskeleton, leukaemia, mechanobiology, nucleus, therapeutic target

## Abstract

Cells sense physical cues from their environment and convert them into biochemical responses through mechanotransduction. Unlike solid tumours, the role of such forces in haematological cancers is underexplored. In this context, immune cells experience dynamic mechanical stimuli as they migrate, extravasate and home to specific tissues. Understanding how these forces shape B‐cell function and malignancy represents a groundbreaking area of research. This review examines the key mechanosensory pathways and molecules involved in lymphocyte mechanotransduction, beginning with mechanosensory proteins at the plasma membrane, followed by intracellular signal propagation through the cytoskeleton, eventually highlighting the nucleus as a ‘signal actuator’. Subsequently, we cover some measurement approaches and advanced systems to investigate tumour biomechanics, highlighting their application in the context of B cells. Finally, we focus on the implications of mechanobiology in leukaemia, identifying molecules involved in B‐cell malignancies that could serve as potential ‘mechano‐targets’ for personalised therapies. This review emphasises the need to understand how lymphocytes generate, sense and respond to mechanical stimuli, which could open avenues for future biomedical innovations.

**Impact statement**

Our review is particularly valuable in highlighting the underexplored role of mechanobiology in B cell function and malignancies, while also discussing emerging techniques that can advance this research area. It bridges mechanotransduction, immunology, and cancer biology in a way that will be of interest to researchers across these three main fields.

## Graphical abstract

B cells sense external mechanical forces and convert them into biochemical signals through mechanotransduction. Understanding how malignant B cells respond to physical stimuli represents a groundbreaking area of research. This review examines the key mechano‐related molecules and pathways in B lymphocytes, highlights the most relevant techniques to quantify and mimic such forces, and discusses their role in haematological malignancies.

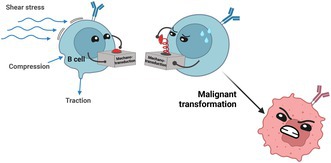

## Abbreviations

**2D**, two‐dimensional

**3D**, three‐dimensional

**AFM**, atomic force microscopy

**AKT**, protein kinase B

**ALL**, acute lymphoblastic leukaemia

**AML**, acute myeloid leukaemia

**APC**, antigen‐presenting cell

**ARP 2/3**, actin‐related protein 2/3 complex

**BCR**, B‐cell receptor

**BCR‐ABL1**, breakpoint cluster region‐abelson

**CAM‐DR**, cell adhesion‐mediated drug resistance

**CD19**, cluster of differentiation 19

**CD34**, cluster of differentiation 34

**CD8**, cluster of differentiation 8

**CK1**, casein kinase 1

**CLL**, chronic lymphocytic leukaemia

**CML**, chronic myeloid leukaemia

**CpG**, cytosine–phosphate–guanine

**DLBCL**, diffuse large B‐cell lymphoma

**ECM**, extracellular matrix

**FAK**, focal adhesion kinase

**FOXO1**, forkhead box protein O1

**HL‐60**, human leukaemia 60

**ICAM‐1**, intracellular adhesion molecule 1

**IgHV**, immunoglobulin heavy‐chain variable region

**IgM**, immunoglobulin class M

**IL‐1β**, interleukin 1 beta

**IS**, immuno synapse

**KD**, knockdown

**KO**, knockout

**LATS1**, large tumour suppressor kinase 1

**LFA‐1**, lymphocyte function‐associated antigen 1

**LMNA**, lamin A

**LMNB**, lamin B

**LMNB1**, lamin B1

**LMNB2**, lamin B2

**LMNC**, lamin C

**MHC**, major histocompatibility complex

**MST1**, mammalian sterile 20‐like kinase 1

**MYO1G**, myosin I gene

**NF‐kB**, nuclear factor kappa‐light‐chain‐enhancer of activated B cells

**NHL**, non‐Hodgkin lymphoma

**NSK**, nucleoskeleton

**N‐WASP**, neural Wiskott–Aldrich syndrome protein

**OS**, optical stretcher

**OT**, optical tweezer

**PA**, polyacrylamide

**PDMS**, polydimethylsiloxane

**PI3K**, phosphoinositide‐3‐kinase

**PIEZO1**, piezo‐type mechanosensitive ion channel component 1

**PM**, plasma membrane

**PYK2**, proline‐rich tyrosine kinase 2

**RT‐DC**, real‐time deformability cytometry

**STK4**, serine/threonine kinase 4

**SUN**, Sad1 and UNC‐84 domain‐containing protein

**SYK**, spleen tyrosine kinase

**TAZ**, WW domain‐containing transcription regulator 1

**TCR**, T‐cell receptor

**TEAD2**, TEA domain transcription factor 2

**TFM**, traction force microscopy

**Treg**, regulatory T cell

**Tyr**, tyrosine

**VLA‐4**, very late antigen‐4

**WASP**, Wiskott–Aldrich syndrome protein

**YAP1**, yes‐associated protein 1

B cells are characterised by the expression of clonally diverse cell surface B‐cell receptors (BCRs), which enable the recognition of antigen epitopes [1]. Their development occurs in distinct stages, beginning in the primary lymphoid organs (bone marrow in adult humans) and continuing with constant recirculation between secondary lymphoid organs (such as the lymph nodes and spleen) and the bloodstream until they encounter a specific antigen. Once this occurs, B cells are retained in the lymph nodes, where they proliferate and differentiate into effector or memory B cells [2]. The continuous recirculation of B cells between lymphoid and nonlymphoid organs relies on the blood vessels and lymphatic system as trafficking routes. This process subjects B cells to ongoing cellular deformation as they squeeze between endothelial cells, exit the bloodstream and rapidly adapt to distinct tissue compartments [1, 3, 4]. Both healthy and malignant B cells undergo recirculation and deformation, though specific differences between them remain under investigation.

B cells experience and respond to a wide range of mechanical forces acting at the cellular, organelle and molecular levels, which influence their behaviour [5, 6, 7]. They do so by converting the mechanical stimuli into intracellular biochemical responses in a process known as mechanotransduction [8]. This process, which is universal to most cell types, is essential for regulating diverse functions such as cell deformation [9], movement [10], stiffness [11], immune response [9, 12], tissue homeostasis and mechanosensing in the tumour microenvironment. Mechanotransduction occurs through a multi‐step process that begins at the plasma membrane, where mechanosensory proteins detect mechanical changes in the external environment [13]. The mechanical signal is then transmitted through the intracellular compartment, leading to cytoskeletal reorganisation [9], gene expression regulation [14], intracellular shuttling [15] and chromatin remodelling [16].

**Fig. 1:**
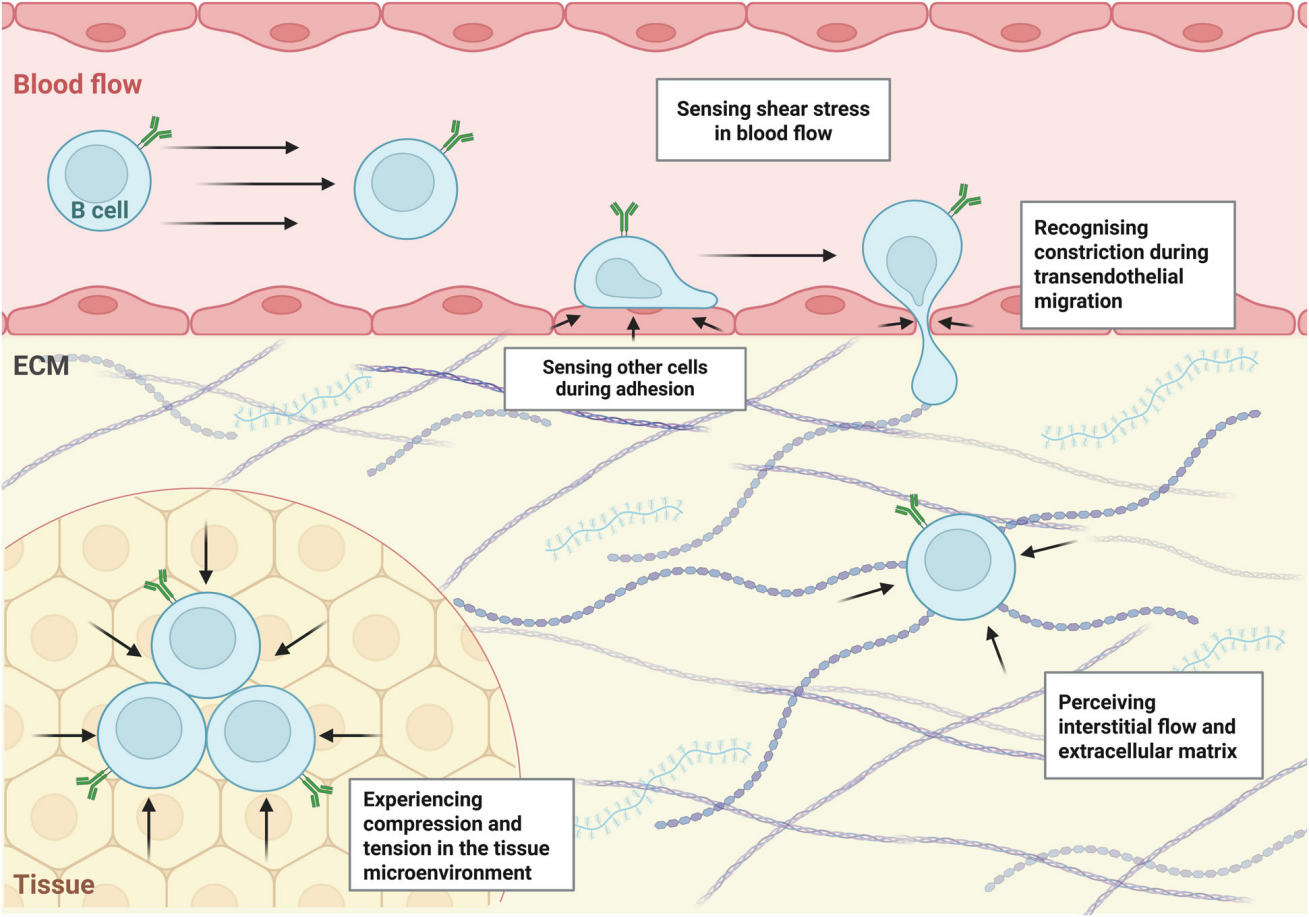
Mechanical forces acting on circulating and extravasating lymphocytes. During circulation in the peripheral blood, B cells are exposed to shear stress generated by blood flow (top). During extravasation into tissues, B cells experience constriction as they migrate out of the blood stream. Within interstitial compartments, B cells encounter hydrostatic pressure and, eventually, the compression and tension generated by the surrounding microenvironment (bottom).

Mechanical stimuli can be broadly categorised into (a) applied forces, which originate from the extracellular environment, and (b) endogenous forces, which arise from within the cell [17]. Regarding the former, B cells are exposed to hydrostatic pressure, shear stress, interstitial flow [18, 19], and both tensile and compressive forces as they traverse tissues and the bloodstream, where they closely interact with their microenvironment [5, 6, 20] (Fig. 1). These fluid shear forces significantly influence B‐cell behaviour and morphology. In circulation, shear stress varies depending on the type of blood vessel, ranging from 10 to 60 dyn/cm^2^ in arteries to 1 to 10 dyn/cm^2^ in veins [21]. In tissues such as the lymph nodes, B cells are also exposed to interstitial flow, where peak fluid shear stress ranges between 0.8 and 1.3 dyn/cm^2^ [22]. The specific effects of these forces on individual B‐cell behaviour, however, remain to be fully elucidated. To accommodate these mechanical forces, B cells leverage endogenous forces to dynamically regulate their shape, elasticity, stiffness, viscosity and friction [7, 23, 24].

The cytoskeleton plays a central role in these adaptations, providing structural support and enabling the mechanical flexibility required for trafficking and function. For example, B cells can reposition their cytoskeleton to detect exogenous antigens, triggering their activation, germinal centre formation and high‐affinity antibody production within the adaptive immune response [25].

In this review, we will provide an in‐depth examination of mechanotransduction in B cells. We begin by exploring the plasma membrane, where key mechanosensory molecules play a critical role in B cells' mechanosensing. From this point, we examine the intracellular mechanisms of mechanical force propagation, focusing on the cytoskeleton and its critical role in force transmission. Moving deeper, we investigate the nucleus and its components involved in the mechano‐response, investigating how these structures contribute to cellular adaptation to mechanical stimuli. Finally, we discuss the principal technologies used to measure cellular mechanical properties and the current *in vitro* approaches employed to study cellular mechano‐adaptation to imposed force. Throughout, we aim to shed light on the connection between B cells' mechanical‐related molecules and their implication in the development of haematological cancers, as well as their potential as therapeutic targets. If no information is available for B cells, we provide examples related to other lymphocytes when appropriate.

## Mechanotransduction in healthy and malignant lymphocytes

Mechanotransduction enables lymphocytes to sense and respond to mechanical forces, influencing their migration, activation and overall function. This section examines the key components of this process in healthy and malignant lymphocytes, beginning with the mechanical properties of the plasma membrane, followed by the role of the cytoskeleton and the nucleus in transmitting and integrating mechanical signals.

### The plasma membrane: from its composition to its mechanosensory properties

**Fig. 2:**
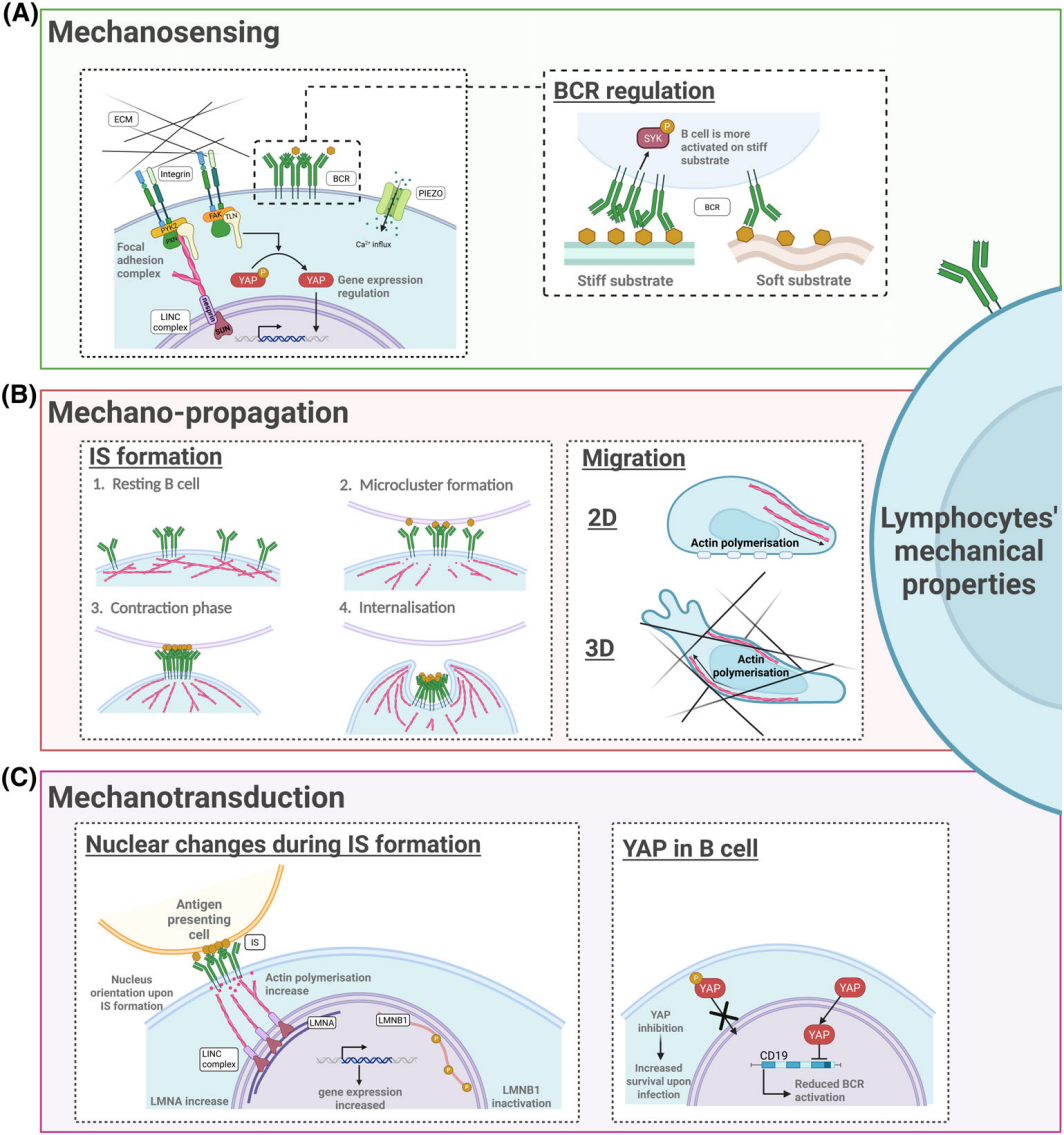
Key components involved in B‐cell mechanosensing, mechano‐propagation, and mechanotransduction. (A) Mechanosensory molecules: the role of the B‐cell receptor (BCR) as a mechanosensor during B‐cell activation. (B) Intracellular mechano‐propagation: the influence of cytoskeletal dynamics in immunological synapse (IS) formation and B‐cell migration in 2D and 3D environments. (C) Mechanotransduction: the influence of the BCR in nuclear orientation, LINC complex function, lamin nucleoskeleton regulation and YAP function.

The plasma membrane (PM) is a dynamic lipid bilayer composed of sphingolipids, phospholipids, cholesterol and proteins, which separate the inner and outer cellular compartments [26]. It forms specialised regions, such as lipid rafts, and maintains fluidity, asymmetry and integrity, enabling key cellular processes including endocytosis, exocytosis, migration and cell division [27, 28]. The mechanical properties of the membrane depend on its specific lipid and protein composition. For example, immature B cells have lower membrane cholesterol levels than their mature counterparts, which was found to be associated with impaired BCR aggregation. As these cells must efficiently recognise and respond to antigens for proper immune activation, this finding suggests that cholesterol content may influence antigen responsiveness [29]. Besides cholesterol, other lipids in the PM were found to rule various cellular processes. For instance, changes in phosphatidylserine distribution within the PM were observed to regulate signal transduction in T cells. Such signals include the downstream activation of IL‐1β and CD62L‐related inflammatory response, as well as lymphocyte migration and adhesion processes [30]. In addition to its composition, the PM is characterised by several mechanosensory molecules, including adhesion molecules, ion channels and receptors [8] (Fig. 2A).

#### B/T‐cell receptor signalling molecules

Receptors on the PM include B‐ and T‐cell receptors (BCR/TCR) that detect specific antigens and trigger lymphocyte development and immune activation [31, 32]. Recent studies have demonstrated that TCR activation depends on mechanical stimuli from the extracellular environment [33, 34]. For example, Blumenthal *et al*. showed that T‐cell priming by antigen‐presenting cells (APCs) is influenced by mechanical signals, whereby the strength of interaction between the TCR and the MHC increases proportionally with APC stiffness [35].

The role of the BCR as a mechanosensor has been less extensively studied than that of the TCR, but it was demonstrated that BCR activation is also influenced by APC stiffness [5]. Wan *et al*. investigated the activation of primary B cells upon binding to antigens tethered to a polydimethylsiloxane (PDMS) gel of varying stiffness. Using super‐resolution microscopy, they quantified the accumulation of BCR and key indicators of early BCR signalling and activation such as phosphorylated spleen tyrosine kinase (pSYK) and phosphorylated tyrosine (pTyr) molecules at the immunological synapse (IS). B cells interacting with a high‐stiffness substrate exhibited increased BCR accumulation and enhanced expression of activation markers compared to those on a softer substrate [36]. The same authors also showed that IgM‐BCR and class‐switched IgG and IgE‐BCR require different thresholds of mechanical energy for activation [37].

Others have since confirmed that stiffer substrates enhance B‐cell activation [38], and that B‐cell proliferation is higher on softer substrates, likely due to BCR engagement and subsequent phosphorylation of PI3K, AKT and FOXO1 molecules. These findings highlight the complexity of mechanosensing and mechanotransduction in B cells. Later, Wang *et al*. used traction force microscopy to establish a direct correlation between the traction force exerted during B‐cell activation and the stiffness of the antigen‐presenting substrate. Moreover, they identified a positive association between traction force strength and BCR micro‐cluster formation [39]. Taken together, these studies highlight a fundamental role of the mechanical forces in regulating B‐cell function. As antigen recognition by B cells is mechanically regulated by the rigidity of the substrate, this aspect could be relevant in the context of vaccine development and diseases caused by hyper‐ or hypo‐activation of B lymphocytes.

#### Integrins

Another important class of mechanosensory proteins is integrins [7], a structurally complex family of molecules essential for cell–cell and cell–extracellular matrix adhesion [40]. Two major integrins are expressed by both B and T cells: leukocyte function‐associated antigen 1 (LFA‐1) and very late antigen 4 (VLA‐4). These integrins have critical roles in leukocyte adhesion, activation and trafficking [41]. Upon extracellular matrix (ECM) protein binding, integrins cluster to form focal adhesions (FAs) multi‐protein complexes that serve as signalling hubs by interacting with adaptor proteins (such as paxillin, talin and vinculin [8]) to facilitate mechanical signal transmission through the cytoskeleton [42]. Focal adhesion kinase (FAK) is one of the first molecules recruited following external stimuli [43], triggering downstream signalling pathways, including Yes‐associated protein 1 (YAP1) [44]. Shaheen *et al*. demonstrated the essential role of FAK in B‐cell mechanosensing, with loss of FAK abolishing substrate stiffness discrimination and activation [45].

#### Ion channels

Ion channels also function as mechanosensory molecules. They facilitate calcium influx, triggering downstream signalling pathways primarily involved in cytoskeletal reorganisation and cell trafficking [46]. Among these, PIEZO1 is one of the best‐characterised and widely expressed mechanosensitive ion channels. PIEZO1 is a heterodimeric protein that detects and responds to external mechanical stretch and shear stress, leading to calcium influx [47]. This channel has been implicated in various physiological and pathological contexts, including T cells' cytotoxic potential and B‐cell activation [48]. Interestingly, researchers have shown that mechanical stress alone is sufficient to activate innate immunity via PIEZO1. This finding is particularly relevant, since mechanical alterations within the microenvironment may generate a mechanical energy sufficient to induce ion channel activation, thereby contributing to disease progression and autoinflammation. Therefore, this mechanotransduction process represents a potential target for therapeutic intervention [15]. Eventually, PIEZO1's role in T‐cell priming, activation and cytokine production has been further explored [49, 50].

A recent study by Kwak *et al*. investigated PIEZO1 in B cells, demonstrating that perturbation of PM tension, such as contact with a solid surface, is sufficient to activate PIEZO1, allowing Ca^2+^ influx. Interestingly, this influx was independent of BCR crosslinking. Instead, it seems that PM mechanosensing via PIEZO1 precedes BCR‐dependent B‐cell activation. The researchers proposed that this mechanism might explain why B cells predominantly respond to membrane‐presented antigens rather than soluble antigens *in vivo* and suggested that it could be relevant to improve vaccine efficacy [51].

### Intracellular mechano‐propagation through cytoskeletal tension

The transmission of mechanical signals, whether driven by extracellular or cell‐generated forces, relies on major cytoskeletal rearrangements, particularly within the actin cytoskeleton [8]. At the cell periphery, beneath the PM, actin is organised into a three‐dimensional network known as the actin cortex. In lymphocytes, this cortex consists of a contractile network of randomly distributed actin filaments and myosin II motor proteins, which generate contractile and tensile forces essential for regulating cell shape, contraction, division and migration [24, 52]. The actin–myosin tension at the leading edge, in conjunction with integrin‐mediated adhesion, generates the propulsive force required for cell movement [53].

#### Cell shape and migration

B cells adopt an amoeboid migration mode, characterised by immature focal adhesions, which allows them to rapidly change shape in a simple two‐dimensional (2D) environment [54]. Interestingly, leukocyte amoeboid migration alone is insufficient for efficient movement through body compartments, suggesting the existence of additional mechanisms at play [55]. Indeed, a study using dendritic cells as a model for interstitial leukocyte migration in a three‐dimensional (3D) environment [56] demonstrated the presence of a retrograde actin flow—a movement of actin from the front to the back of the cell body—during migration [57]. This retrograde flow generates friction with the surrounding microenvironment, where topographical features have a major role in force transmission during adhesion‐free migration [53, 56].

Studies on T cells suggest they can switch migration modes and speeds via LFA1/ICAM‐1 interactions [58]. Notably, LFA‐1 has been proposed to act as a frictional interface in confined spaces, enabling cell locomotion without adhesion [59]. A similar mechanism has been proposed for B cells, but data on these process remain limited [53]. These findings highlight the reciprocal relationship between cells and their microenvironment, a phenomenon commonly referred to as ‘mechanoreciprocity’ [60].

#### BCR activities

The actin cytoskeleton also plays a direct and versatile role in modulating antigen receptor activity, bridging physical and biochemical responses and facilitating major cellular deformations (Fig. 2B) [61]. In resting B cells, BCRs exist as monomers within a confined space, with lateral motility restricted by the actin cortex [62]. Transient actin depolymerisation and detachment of the actin cortex from the plasma membrane are required for BCR micro‐cluster formation, which initiates the signalling cascade [63]. Inhibiting actin polymerisation (using latrunculin B) leads to spontaneous BCR micro‐clustering and activation, whereas inducing actin polymerisation (using jasplakinolide, an inducer and stabiliser of pre‐existing actin filaments) has the opposite effect [64].

To increase the number of these signalling hubs, the B‐cell spreads out its membrane across the presenting cell in a process named cell spreading, enabling contact with a greater amount of antigen [65]. While B cells spread, actin undergoes significant reorganisation. The contact zone between the B‐cell and APC requires ARP2/3 complex activation via WASP and/or N‐WASP proteins. Knockout of these proteins has been shown to reduce cell spreading, confirming their role in this process [66]. The stretch of the cell spreading depends on the affinity and number of antigen on the APC. Interestingly, by using a platform with immobilised ligands and tuneable mechanical forces, Wan *et al*. observed that higher mechanical forces generally cause more vigorous cell spreading and enhance BCR accumulation at the IS. This suggests that the mechanical properties of the APC strongly regulate BCR activation and that BCR‐IgM alone could function as a mechanosensor, as previously discussed [37]. Once maximal spreading is achieved, the B‐cell initiates a contraction phase, during which retrograde actin flow, driven by myosin II contraction, promotes BCR micro‐cluster movement towards the centre of the contact area [67]. Inhibition of myosin II activity impairs IS formation and BCR signalling [52].

Following antigen binding, the BCR complex undergoes internalisation through various actin‐dependent mechanisms, which are mechanically demanding processes requiring actin polymerisation [68]. The most well‐characterised pathway is clathrin‐mediated endocytosis, which has been proposed to be a mechanism depending on membrane tension [69]. Clathrin knockout significantly impairs BCR internalisation. However, it has also been demonstrated that B cells can internalise antigen‐specific BCRs through a clathrin‐independent mechanism that still requires actin remodelling [70]. Recent insights suggest that the switch between these internalisation modes is determined by the size of BCR clusters [71].

### The mechano‐actuator property of the nucleus

The mechanical signals detected by mechanosensory molecules at the PM are transmitted to the nucleus via cytoskeletal modifications. Recent evidence suggests that the nucleus does not only receive signals from the cytosol but also functions as a mechanosensor in its own right [72]. Given that the nucleus is the largest and stiffest organelle, occupying most of the cell volume in B cells (Fig. 2C) [1, 7], and it serves as the primary constraint in cellular deformation. Emerging studies, mostly conducted in 3D models, propose the nucleus to function as a mechano‐gauge, meaning that nuclear envelope stretching in confined environments correlates with specific cellular behaviours [73, 74].

Research on amoeboid‐migrating cells, such as dendritic cells and T cells, indicates that they prefer larger pores and navigate towards the path of least resistance. Notably, these cells move in a nucleus‐first configuration, with the nucleus guiding migration through constrictions [75]. Whether B cells adopt a similar mechanism remains unknown, as no studies have investigated this phenomenon yet.

#### Protein shuttling as mechanotransducer

Studies on adherent cells have identified the nuclear entry of proteins in response to mechanical stimulation, such as β‐catenin [76], zyxin [77] and paxillin [78], which regulate gene expression. Nuclear mechanotransducers, including the homologous transcriptional coactivators YAP1 and WW domain‐containing transcription regulator protein 1 (WWTR1/TAZ), have also been identified [79].

In B cells, YAP1 and TAZ seem to be maintained at lower expression levels than in other human cell types [80], for yet unknown reasons. Interestingly, new insights into the immunosuppressive activity of regulatory T cells (Treg) have shown that YAP1 deregulation enhances Treg activities [81]. In B cells, deregulation of YAP1 and TAZ could have a similar role by regulating B‐cell activities; however, the exact role of these proteins in B cells remains to be elucidated. Xiaoming *et al*. investigated the regulation of CD19, a B lymphocyte antigen and biomarker involved in BCR regulation, by MST1 kinase, a serine/threonine‐protein kinase, key component of the Hippo signalling pathway and a pro‐apoptotic factor. They found that MST1 negatively regulates YAP1. In the absence of MST1, YAP1 translocates to the nucleus, where it represses CD19 transcription through TEAD2 activation, highlighting its potential role in B‐cell activation [82]. Another study demonstrated that phosphorylation‐mediated inhibition of YAP1 promotes B‐cell survival by suppressing IL‐1β secretion during *Salmonella* infection, thereby facilitating bacterial dissemination and persistence [83].

#### The nucleoskeleton function

Beyond protein translocation, a direct physical connection between the nucleus and the PM has been identified, mediated by the Linker of Nucleoskeleton and Cytoskeleton (LINC) complex [84].

This complex consists of nesprin, which binds directly or indirectly to the cytoskeleton, and SUN, which interacts with the nucleoskeleton (NSK) [8], a complex network of interacting proteins with lamins as core structural elements [85]. The two major lamin isotypes are lamin A (LMNA) and lamin B (LMNB), with lamin C (LMNC) arising from alternative splicing of LMNA. A recent study revealed that during IS formation, B cells reorient their nucleus towards the antigen contact site. Silencing either nesprin or SUN impaired nuclear repositioning and IS formation, leading to defective BCR internalisation [86]. Further research using magnetic tweezers to apply force directly to nesprin in isolated nuclei demonstrated that nuclear stiffening occurs via lamins [84, 87].

LMNA is a key determinant of nuclear shape and stiffness; cells lacking LMNA exhibit softer nuclei and increased nuclear blebbing [88, 89]. *Lmna* knockout mice die shortly after birth, displaying severe defects in T‐ and B‐cell development, as well as reduced thymus and spleen sizes [90].

LMNB, encoded by two different genes (*LMNB1* and *LMNB2*) [91], is expressed at varying LMNA:LMNB1 ratios depending on tissue type. In human haematopoietic stem cell progenitors from bone marrow, LMNA expression is low and the low LMNA:LMNB1 stoichiometry is thought to affect nuclear stiffness and deformability [92, 93]. For example, low LMNA facilitates neutrophil deformation, aiding their passage through microporous barriers [94, 95].

Unlike other cell types, lymphocytes do not maintain stable LMNA expression; rather, LMNA levels fluctuate during differentiation, activation and migration [96]. González‐Granado *et al*. reported that resting naïve T cells have minimal LMNA expression, but following TCR activation, LMNA levels rise significantly, promoting IS formation, likely through actin polymerisation [97]. Further studies are required to determine whether a similar mechanism may exist in B cells.

Indeed, few studies have examined lamin organisation and function in B cells. Sherif *et al*. investigated migration in a subtype of diffuse large B‐cell lymphoma (DLBCL) and found that LMNA/C expression was elevated in both primary cells and cell lines, impairing their ability to deform and migrate through tight spaces [98]. Another study reported that during B‐cell activation and germinal centre formation, chromatin binding to LMNB1 decreases, likely due to its phosphorylation, which promotes gene expression. Chromatin immunoprecipitation sequencing revealed that the released DNA corresponds to kappa and heavy variable immunoglobulin domains, linking LMNB1 regulation to somatic hypermutation [16].

## Probing lymphocyte mechanics: from measurement techniques to biological implications

Understanding the key players involved in cellular mechanotransduction is crucial for deciphering how cells sense and respond to mechanical stimuli, influencing functions such as migration, activation and survival. It is also essential, however, to consider the intrinsic mechanical properties of cells, which determine their ability to deform in response to mechanical forces over time. Indeed, changes in cell stiffness have been reported in various physiological and pathological conditions, including ageing [99], infection [100] and cancer [11, 101]. Given the biological significance of these mechanical properties, there is a growing need for advanced techniques to study and quantify them at high resolution.

### Measurement techniques

**Fig. 3:**
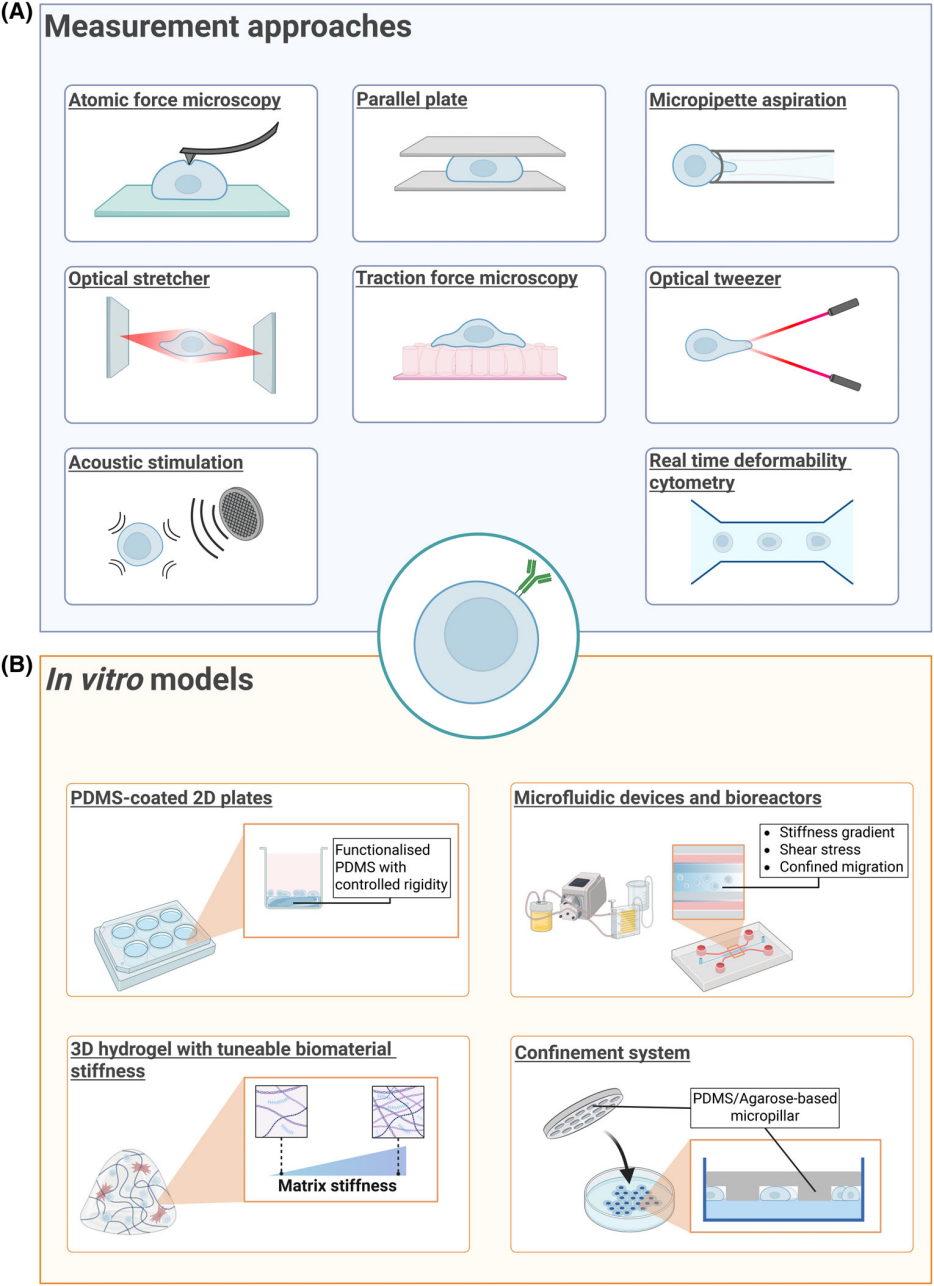
Experimental approaches for investigating lymphocytes' mechanical properties. (A) Technologies to measure single‐cell mechanical parameters, such as Young's Modulus, viscosity and deformability. (B) Models for mechanical stimulation, including *in vitro* systems to expose cells to mechanical forces and microenvironmental conditions.

Over the past two decades, numerous experimental methods have been developed to study and quantify the different forms of cellular deformation. In this section, we outline several of these methods (Table 1) and point out their application to better understand B‐cell mechanotransduction in both physiological and pathological conditions. While some are not established for B cells yet, they have been successfully used for other lymphocytes, making them potentially relevant for B‐cell research (Fig. 3A).

**Table 1:** Measurement methods used to study and quantify lymphocyte mechanical properties, along with their corresponding advantages and limitations.

Method	Applied to B cells	Measured outcome	Advantages	Limitations	References
Atomic force microscopy (AFM)	Yes	Cell stiffness, elasticity, viscoelasticity, deformability	High‐resolution, single‐cell analysis, real‐time monitoring, suitable for heterogeneous samples	Direct contact may alter intrinsic mechanical properties, possible tip‐induced artefacts, limited scan area, limited depth profiling, complex data interpretation	[102, 107]
Parallel plate	No—applied to T cells and other leukocytes	Stiffness, viscosity, deformability	Precise control, simple set up, wide range of material, small sample size	Edge effects, no high‐throughput, slippage issue, no vertical deformation	[110, 111]
Traction force microscopy	Yes	Cell traction force	Noninvasive, high spatial resolution, real‐time measurements, force‐mapping generation, insight into cell–environment interactions	Complex data analysis, limited force range, deep limitations penetration at microscope, possible substrate artefacts	[112, 113, 114, 115]
Optical stretching (OS)	No—applied to other leukocytes	Deformation, viscosity, stiffness	Noninvasive, label‐free, noncontact nanoscale force application, high precision, real‐time monitoring	Limited to small sample usage, limited focus range, heating, possible optical artefacts	[110, 117, 118, 119, 120, 121, 122]
Optical tweezer (OT)	Yes	Stiffness, elasticity, deformability, optical trapping force, adhesive forces	Noninvasive, label‐free, noncontact piconewton force application, high precision, high spatial resolution, single subcellular structure tracking	Limited force range, complex set up, possible laser‐induced damage, limited to small isolated samples	[110, 116, 117, 118, 120, 122]
Acoustic field‐based approaches	No—applied to other leukocytes	Morphological parameters, compressibility, viscoelastic properties	Noninvasive, label‐free, noncontact, measurement in suspension, measurement of 2D and 3D sample, real‐time, dynamic measurement, high spatial resolution	Limited depth resolution, complex modelling for data interpretation, better with homogeneous material	[123, 124, 125, 126, 127]
Micropipette aspiration	No—applied to T cells and other leukocytes	Stiffness, morphological parameters, viscosity, shear thinning coefficient	Single‐cell study, real‐time monitoring, easy to implement, high spatial resolution at single‐cell level	Low‐force measurements, difficult for large and stiff samples, high aspiration pressure may cause unnatural deformation	[128, 129, 130, 131, 132]
Real‐time deformability cytometry (RT‐DC)	Yes	Morphological parameters, rheology, cell size, stiffness	Noninvasive, label‐free, noncontact, high‐throughput, single‐cell resolution, quantitative, volume sample versatility (from small to large)	Requires specialised equipment, possible flow‐induced stress, limited cell structural information	[133, 134, 135, 136, 137, 138]

#### Atomic force microscopy

Atomic force microscopy (AFM) is widely regarded as the gold standard for studying the mechanical properties of tissues and individual cells [102]; it enables the measurement of both elastic [103] and viscous cellular responses [104, 105]. AFM operates using a scanning probe microscope, in which a scanning tip, mounted at the end of a flexible cantilever, probes the sample's surface [106]. As the tip moves across the sample, the cantilever's deflection changes, reflecting the specimen's mechanical properties [107].

In single‐cell analysis, the contribution of different cellular structures to mechanical properties depends on the applied force. For example, in our previous work on healthy and malignant B cells, we indented lymphocytes by 500 nm to assess the role of the actomyosin cortex in cellular stiffness and to investigate whether chemotherapy agents could alter their mechanical properties. Interestingly, we found that the actomyosin complex architecture and contractility of chronic lymphocytic leukaemia (CLL) cells correlate with specific mechanical features that are different from those of healthy B cells [11]. In another study, researchers used AFM to measure changes in lymphoma B‐cell mechanics following chemotherapy treatment. They observed a significant reduction in B‐cell lymphoma stiffness upon treatment, demonstrating that AFM can be a valuable tool for improving the understanding of chemotherapy's effects [108]. More recently, in collaboration with other researchers, we optimised a protocol to produce a wedge cantilever. This new geometry overcomes the limitations arising from the characteristics of our sample (small, roundish and soft cells), enabling the measurement of the stress–relaxation function of CLL cells, which was difficult to assess using more traditional tips, such as square‐based pyramidal or colloidal tips [109].

#### Parallel plate

The parallel plate approach is a technique similar to AFM that applies force to the nonadhesive side of the cell. In this method, a cell is placed between two parallel plates—one flexible and one rigid—allowing for the assessment of Young's modulus, deformability‐relaxation and creep function [110].

This method has been used to investigate the viscoelastic properties of T cells and myeloid APCs in both resting and inflammatory states. The findings demonstrated that T cells encounter varying cell rigidities when interacting with APCs, and that these mechanical properties can change during inflammatory processes [111]. This method has not been applied to B cells so far, but we believe that this approach could be used to extract information on how B‐cell stiffness changes during activation or inflammatory processes.

#### Traction force microscopy

Traction force microscopy (TFM) quantifies cellular traction forces—the force exerted by a cell to stretch or pull its surroundings—by optically measuring substrate deformations caused by cell‐generated forces [112]. To quantify the substrate deformations, the setup uses an adhesion array that typically consists of a soft gel embedded with beads or a micropillar array. The deflection of a given pillar or bead, which can be measured through optical imaging, depends solely on the force applied to that specific pillar or bead by the cell [113]. TFM has been adapted for both adherent and suspension cells and has also been optimised for measurements in 3D matrices [112]. In B cells, TFM has been employed to investigate the forces exerted on the adhesion substrate during activation. In particular, Kumari *et al*. optimised a method to investigate the spatial and temporal distribution of the force field generated by the BCR during antigen extraction. In their protocol, the substrate was adapted to mimic the rigidity of the physiological environment and to allow the measurement of several cells simultaneously. Interestingly, they observed that mechanical forces are spatio‐temporally patterned at the IS, with an interplay between peripheral and central forces, both governed by the actomyosin complex [114, 115].

#### Optical‐based approaches

Two major optical field‐based methods for measuring cell mechanical properties are the optical stretcher (OS) and the optical tweezer (OT) [110, 116]. These noninvasive techniques rely on focused laser beams to manipulate micro‐scale objects, such as cells or subcellular components [117, 118]. OS uses two high‐power diverging laser beams to trap and deform an entire cell. Increasing laser power enhances the applied force and cellular deformation [119], but may also induce cell damage or heating, potentially altering measurements [120].

A study investigating neutrophils and red blood cells introduced a linear optical stretcher as a potential high‐throughput method for assessing cellular mechanical properties. The study proposed a microfluidic system incorporating an optical stretching region, where individual cells are captured and deformed directly within the flow. This approach enables cell discrimination based on their deformation [121]. By contrast, OT employs a focused laser beam to apply both attractive and repulsive forces on the specimen [122]. While this method has a limited force range and cannot fully deform a cell, it is particularly useful for studying local mechanical events [117]. For example, Duś‐Szachniewicz *et al*. used OT to measure the initial force of interaction between normal and malignant B cells and their microenvironment. They developed a method that allowed for the discrimination of normal B cells from non‐Hodgkin lymphoma (NHL) B cells based on their adhesion to mesenchymal stromal cells. Their findings showed that malignant B cells exhibit significantly reduced adhesive properties compared to normal B cells [120].

#### Acoustic field‐based approaches

Acoustic field‐based techniques provide a label‐free, contactless and biocompatible method for cell manipulation [123]. These methods have been applied in various contexts, including cell separation based on size and mechanical properties. For example, Ding *et al*. developed a noninvasive approach for sorting similarly sized cells based on their compressibility. Using this method, they successfully separated circulating human breast cancer cells from nonmalignant leukocytes [124]. Acoustic fields have also been employed to measure cellular compressibility [125]. A particularly interesting approach is Brillouin microscopy, which relies on light scattering due to interactions between light and gigahertz acoustic waves, a process known as Brillouin light scattering. This technique provides insights into the viscoelastic properties of biological samples [126]. It is a noninvasive, label‐free method that can also be applied to 3D samples [127]. To date, no studies have explored the application of these methods to B cells. However, Brillouin microscopy could serve as a valuable tool for studying the elastic modulus and viscoelastic response of B cells in suspension, without the need to induce cell adhesion.

#### Micropipette aspiration

Micropipette aspiration is one of the earliest techniques developed for measuring single‐cell mechanical properties. In this method, a suspended cell is aspirated into a micropipette by applying controlled negative pressure [128, 129]. The cell position and morphological changes at the micropipette tip are then tracked over time using a microscope. This approach enables the measurement of stiffness, cell volume, viscosity and the shear thinning coefficient [130]. In a recent study, micropipette aspiration was used to examine mechanical changes in T cells during activation. The findings revealed that activated CD8^+^ cells, compared to naïve CD8^+^ cells, exhibit increased volume, cortical tension and initial elastic response, which the authors proposed might contribute to spontaneous motility [131]. Micropipette aspiration has also been applied to mimic the squeezing and deformation of T cells passing through capillaries or during transendothelial migration, in order to understand whether a regulatory mechanism exists that prevents membrane rupture during large deformations. Interestingly, this approach revealed that, during aspiration, T cells maintain a constant volume while their surface area increases, and that membrane rupture occurs only when the membrane area expansion exceeds a critical threshold, which is independent of the aspiration by the micropipette [132]. Currently, micropipette aspiration has not been applied to B cells, but it could be used to study, for example, B‐cell migration in constricted spaces and the associated mechanical properties.

#### Real‐time deformability cytometry

Real‐time deformability cytometry (RT‐DC) is a high‐throughput method for investigating cellular morphology and rheology—deformation of matter [133]—at the single‐cell level. Cells are exposed to laminar flow in a microchannel with dimensions comparable to cell size, leading to cell deformation without direct mechanical contact [134]. This technique allows for the acquisition and segmentation of images of deformed cells, providing information on cell size, deformability and stiffness [135, 136]. RT‐DC is particularly sensitive to alterations in the cytoskeleton. For example, in a study by Golfier *et al*. on the leukaemia cell line HL‐60, it was demonstrated that actin cortex integrity and microtubule networks are dominant factors in determining cell deformation [137]. In another work, RT‐DC was used to uncover Young's modulus and viscosity in high‐throughput screening of 100 cells per second. Moreover, the approach allows for the discrimination of different leukocyte lineages, such as B cells and CD4^+^ T cells [138]. The number of studies on isolated B cells is limited; however, this gap could represent an opportunity for further research.

### *In vitro* systems

Alongside advances in techniques to measure and quantify cellular mechanical properties, modelling cell biomechanics is emerging as a promising approach to understanding cellular mechanobiology. Recent progress in bioengineering has led to a wide range of *in vitro* systems for investigating the effects of mechanical stimulation on cells, ranging from standard 2D cultures to 3D systems and microfluidic devices [139]. In this section, we highlight examples of these systems, with a particular focus on B‐cell applications (Table 2, Fig. 3B).

**Table 2:** *In vitro* models for investigating the effects of mechanical stimulation on cells of interest, listed together with their corresponding advantages and limitations.

Model	Applied to B cells	Mechanobiological force encompassed	Advantages	Limitations	References
2D culture with variable rigidities	Yes	Matrix stiffness	Low cost, easy‐to‐use, high‐throughput studies	Over‐simplified model, lack of 3D environment, limited representation of mechanical forces, inaccurate cell morphology	[38, 45, 140, 141, 144]
3D hydrogel with tuneable biomaterial stiffness	Yes	Matrix stiffness, tensile‐compressive stress, cell‐ECM adhesion, traction forces, viscoelasticity of the surrounding environment	Biomimetic environment, control over mechanical cues, cell morphology	Material selection, limited reproducibility, difficulty in tuning properties overtime, lack of standardised protocols, readout optimisation	[147, 148, 149, 172]
Microfluidic devices and bioreactors	Yes	Shear stress, interstitial flow, hydrostatic pressure, tensile‐compressive stress, stiffness, cell deformation	Dynamic mechanical and fluidic stimulation, control of nutrient and oxygen delivery, scalability, real‐time monitoring	Complexity in the experimental design and fabrication, costs, low reproducibility, limited scalability, technical skills and expertise requirement	[150, 153, 154, 155, 156, 157, 158, 159, 160, 161, 162]
Cell confinement systems	Yes	Cell deformation, confinement‐induced migration, compressive stress	Mimicking of different strengths of confinement, control over cell shape and morphology, possibility to collect cells after confinement, real‐time monitoring	No standardised protocols for cells in suspension, edge effect, low reproducibility, possible slippage issues, difficulty of integrating with other experimental settings, limited scalability, limited control	[10, 54, 158, 164, 165, 166]

#### 2D culture plates with functionalised PDMS/agarose

One well‐established system involves designing 2D cultures in which cells adhere to a PDMS layer, functionalised to exhibit different rigidities. Mechanistically, functionalised PDMS is generated by mixing a vinyl‐terminated base agent with a curing agent (methyl hydrogen siloxane). By modulating the ratio between these components, it is possible to obtain surfaces with different stiffness values [140, 141]. Zeng *et al*. employed this system to demonstrate how antigen recognition on a stiff PDMS surface triggers the accumulation of BCR and pSyk molecules at the B‐cell surface [38].

Other mechanobiology studies have leveraged polyacrylamide (PA)‐based gels, formed by polymerisation of a Bis‐acrylate crosslinker and acrylate monomers [142, 143]. Using this method, Shaheen *et al*. investigated how substrate stiffness affects B‐cell activation. By employing an antigen‐labelled artificial PA‐based substrate, they demonstrated that substrate rigidity influences B‐cell activation via the modulation of protein kinase C‐β and FAK [45]. While these models provide easy‐to‐use tools for exploring the mechanical regulation of cell behaviour, they lack the 3D complexity and microarchitecture of the native tissue environment [144].

#### 3D hydrogel with tuneable biomaterial stiffness

This system utilises 3D hydrogels with adjustable biomaterial stiffness, allowing cells to interact with both each other and their environment in a physiologically relevant setting. More specifically, this platform can help to mimic the biochemical and physical properties of the native ECM directly produced by stromal cells in the microenvironment, an aspect that cannot be implemented in 2D culture systems. These hydrogels can be composed of various biomaterials, including alginate, gelatine, silk fibroin and polyethylene glycol. By adjusting the component ratio, viscosity and crosslinking methods, cell‐compatible hydrogels with tailored rigidity can be produced [145, 146]. This system was employed by Zhong *et al*. to generate cell‐laden 3D hydrogels mimicking the lymphoid microenvironment. Using synthetic hydrogels functionalised with adhesive peptides and exhibiting a lymphoid organ‐like stiffness, they encapsulated peripheral blood mononuclear cell‐derived organoids. In doing so, the researchers found that B cells formed compartmentalised germinal centres and exhibited B‐cell reprogramming patterns, including somatic hypermutation and class switch recombination events, enabling the testing of B‐cell responses to lymphoma therapies [147].

In a similar study, B cells were incorporated into a synthetic hydrogel supplemented with microenvironment‐derived components. Compared to conventional 2D culture models, this approach resulted in increased B‐cell survival, enhanced plasma cell differentiation and high‐affinity antibody production [148]. These models are now widely employed due to their cost‐effectiveness compared to animal models and their ability to better mimic the microenvironment and the mechanical cues that influence cell behaviour [149]. Nevertheless, these platforms can be difficult to establish, and the readout strategies require further optimisation.

#### Microfluidic devices and bioreactors

Another *in vitro* platform for studying mechanobiology involves the use of bioreactors and microfluidic devices [150, 151, 152, 153]. These systems allow for the investigation of shear stress by connecting to a pump to generate flow [154] and interstitial fluid pressure effects [155], resembling those experienced by cells in blood and lymphatic vessels. Additionally, these platforms permit studying the impact of stiffness gradients [156, 157] and cellular confinement [158] experienced by cells during transendothelial migration or in tissues. These forces are of fundamental importance in the context of lymphocyte recirculation [7].

Microchannels have been widely used to study cell migration [159] and deformation [160] at single‐cell resolution. For example, in a study on T cells, researchers used a microfluidic device with a fixed 3 μm constriction to examine cell and nuclear deformation during transendothelial migration. They identified formin‐like 1 protein as a key promoter of T‐cell migration and nuclear squeezing [161]. In another study, researchers optimised B lymphocyte binding on a functionalised PDMS layer inside a microfluidic chamber. This system enabled the investigation of shear force effects on B‐cell viability and phenotypic modifications [162]. Besides the enormous advantages of these technologies in helping to decipher the outcome of mechanical stimulation, some limitations must be considered. Most of the microfluidic devices nowadays are home‐made systems, not meeting the need of standardised platforms to translate findings into clinical practice. Moreover, in line with the previous section, readout strategies have to be optimised and adapted to the system employed [163].

#### Cell confinement systems

Cell confinement systems represent another valuable technology for complex mechanobiology studies, and several systems have been developed to investigate how spatial constriction affects cellular behaviour [164, 165]. Mechanistically, these systems confine cells between two parallel glass surfaces at a defined height, controlled by a micropillar‐based system, typically composed of PDMS or agarose on a glass slide [158, 166]. This platform is particularly relevant to lymphocyte mechanobiology, as it allows researchers to mimic the confined local environment that lymphocytes encounter during migration. Additionally, these systems can be integrated with other platforms in which the cell adhesion rate can be tuned by functionalising the adhesion substrate [165]. While this approach offers an interesting tool for studying lymphocyte adaptation to confined spaces, few studies have explored its application in B and T cells, mainly due to technical limitations, complexity in experimental design due to the plasticity of B and T cells, and their low adhesion behaviour, as well as the lack of standardised protocols. One notable study by Caillier *et al*. examined primary T‐cell migration in a PDMS‐based confinement system. Their findings showed that T cells required small and dynamic focal adhesions to move efficiently through the confined space [10]. Čada *et al*. did use an agarose‐based system to confine B cells and investigate their amoeboid‐like migration using live‐cell imaging techniques, dissecting the role of casein kinase 1 (CK1) inhibition in B‐cell migration impairment [54].

### Mechanobiology implication in haematological cancer cells' response to drugs

Cancer has traditionally been considered a disease primarily driven by genetic alterations that affect proliferation, differentiation and cell death [167]. However, advances in measurement approaches and *in vitro* models have since clarified that cancer cells both experience and exploit altered mechanical cues. Indeed, tumours exhibit abnormal stiffness, disrupted blood vessel permeability, and altered fluid pressure, all of which modify the tumour microenvironment. As tumours grow, changes in tissue architecture disrupt homeostasis, facilitating disease progression [168]. In this section, we explore the interaction between mechanical forces and biological processes in haematological cancers, with a particular focus on their influence on cancer cell behaviour and treatment outcomes [169].

#### Mechanotransduction and drug resistance

The interaction between a tumour and its surrounding environment has been identified as a key determinant of drug resistance [143]. For example, Apoorva *et al*. placed DLBCL cells inside a micro‐reactor platform with uniform distribution of fluid flow, which exposes all cells to the same biophysical forces. Their study demonstrated that altered shear stress and nutrient transport, driven by increased vascularisation in lymphoma, promote B‐cell proliferation, upregulation of SYK signalling and modulation of integrins and the BCR. Specifically, they observed how the fluid shear stress can act as an important biophysical stimulus with direct consequences on DLBCL onset, progression and therapeutic response [19].

Despite these insights, knowledge of the mechanical traits of haematological cancers remains limited. While it is well established that circulating and infiltrating haematological malignant cells are exposed to a broad range of mechanical stimuli from their external environment (Fig. 1), the role of these mechanical alterations in tumour progression and drug resistance remains poorly understood [170]. For example, a study on myeloma cells found that cells adhering to a fibronectin‐coated surface were significantly more resistant to apoptosis following standard chemotherapy than those in suspension. Additionally, the authors observed a correlation between integrin ɑ4 expression and doxorubicin resistance, thus hypothesising a cell adhesion‐mediated drug resistance (CAM‐DR) phenomenon. Nevertheless, the exact mechanism involved in this pro‐survival signal is not elucidated yet [171]. Using a 3D hydrogel‐based model, another group demonstrated that matrix stiffness influences the proliferation and drug response of certain acute myeloid leukaemia (AML) cell subtypes [172].

Moreover, intracellular mechanical properties, particularly stiffness and viscoelasticity, are known to affect leukaemic cell behaviour [7]. We recently demonstrated that CLL cells exhibit a less contractile actomyosin complex and reduced stiffness compared to healthy B cells. By treating cells with Bruton tyrosine kinase inhibitors, the primary drugs used in CLL treatment, such as ibrutinib, we observed a partial restoration of actomyosin complex organisation and stiffness, resembling that of healthy B cells, both *in vitro* and *in vivo*. These findings suggest that cortical stiffness is a target of ibrutinib and might be involved in a possible mechanism of drug resistance [11].

Similarly, a study by Lam Wilbur *et al*. showed that acute lymphoblastic leukaemia (ALL) and AML cells exhibit a two‐order magnitude increase in cell stiffness following standard chemotherapy, impairing their ability to pass through microfluidic channels [173]. Others have also reported that cancer cells exhibit altered mechanical properties compared to their healthy counterparts. In CD34^+^ haematopoietic cells isolated from both healthy donors and leukaemia patients, researchers observed that cancerous CD34^+^ cells became stiffer and underwent brittle failure when subjected to external force, whereas healthy CD34^+^ cells maintained their structural integrity [174]. Additionally, a microfluidic single‐cell study on ALL cells treated with standard chemotherapy for 7 days found that treated cells exhibited increased deformability, further supporting the link between cell mechanics and treatment response [175].

#### Molecular regulators of cancer cell mechanics

Recent studies have implicated the deregulation of specific proteins and pathways that govern cancer cell responses to mechanical stress [176]. One well‐established pathway in this context is the Hippo signalling pathway. A 2018 study evaluating Hippo signalling pathway gene expression revealed that TAZ is deregulated in patients with chronic myeloid leukaemia (CML) who are refractory to the tyrosine kinase inhibitor (TKI) imatinib [177]. Others have shown that knockdown of YAP1 impairs leukaemic cell proliferation and activates apoptotic pathways in both CML cell lines and Jurkat leukaemia cells. In addition, YAP1 deregulation in CML was associated with enhanced TKI efficacy *in vitro* [178, 179]. Interestingly, YAP1 has been identified as a tumour suppressor gene in haematological cancers, where it is preferentially inactivated, suggesting that restoring YAP1 function could represent a novel therapeutic strategy [180]. To date, the role of YAP1 in B‐cell malignancies has been limited to a few studies. By examining YAP1 expression in a public dataset, Zhou and colleagues correlated its expression with disease progression and poor prognosis in patients with DLBCL. Moreover, CRISPR/Cas9‐aided knockdown of YAP1 was found to elicit an antitumour effect by regulating the cell cycle, either *in vitro* or *in vivo* [181]. In a similar study, researchers examined YAP1 expression in B‐ALL cell lines and found that upregulation of YAP1 and low phosphorylation levels were associated with worse outcomes, in line with the previous findings. Additionally, they observed that YAP1 knockdown inhibited cell proliferation, thereby confirming its role as a potential oncogene. Furthermore, they identified LATS1—large tumour suppressor kinase—as a potential inhibitor of YAP1 phosphorylation, suggesting that targeting this pathway with verteporfin may improve the prognosis of B–ALL patients [182].

Beyond Hippo signalling, recent studies indicate that lamin deregulation may influence B‐cell leukaemia progression [183]. It has been proposed that LMNB1 exerts protective functions, preventing chromosomal aberrations during clonal evolution in CLL [16]. Specifically, CLL patients with a mutated IgHV chain, which correlates with a favourable prognosis, exhibit increased LMNB1 expression. By contrast, CLL patients with unmutated immunoglobulin genes, which are strongly linked to cytogenetic abnormalities and poor prognosis, show decreased LMNB1 expression [16]. Additionally, hypermethylation of CpGs in the *LMNA* promoter, which silences LMNA/C expression, was found to be a predictor of poor outcome in patients with large B‐cell lymphomas [184].

Integrin‐mediated survival signalling represents another key mechanism promoting B‐cell malignancy progression and therapy resistance [185]. Astier *et al*. demonstrated that stimulation with extracellular matrix‐derived proteins activates integrin β1, which prevents caspase‐3 and caspase‐7 activation, thereby protecting B‐ALL cells from apoptosis [186]. Similarly, another study showed that direct contact between primary CLL cells and bone marrow‐derived stromal cells enhances CLL cell survival by preventing apoptosis [187].

The role of focal adhesion in leukaemia progression has also gained increasing attention. A study by Sonoda *et al*. demonstrated that FAK overexpression in the HL‐60 leukaemia cell line enhances apoptosis resistance by activating the PI3K/AKT and NF‐κB pathways [188]. In 2022, our laboratory identified upregulation of the FAK homologue proline‐rich tyrosine kinase 2 (PYK2) in malignant B cells from CLL patients compared to healthy B cells. Notably, concomitant downregulation of FAK was linked to a transition from stable disease to a more aggressive phenotype. These results suggest that FAK and PYK2 may serve as potential therapeutic targets in CLL, offering a novel strategy aimed at modulating the mechanical properties of cancer cells and their microenvironment [189]. Consistent with this, defactinib, a FAK inhibitor, has emerged as a promising anticancer therapy for various solid tumours [190].

A final, emerging area of interest is the role of myosin in leukaemic cell migration. A study focusing on ALL dissemination and relapse, which is usually associated with poor prognosis, identified *MYH9* as a possible promoter. By injecting ALL cells knocked down (KD) for MYH9 into mice, they observed a slower rate of leukaemia progression and a prolonged survival. This results from an impairment in leukaemia engraftment, especially at the level of the central nervous system. The study suggests that this effect might be correlated with an impaired migration ability and an inefficient transendothelial migration process in MYH9 KD ALL cells, pointing out MYH9 as a potential therapeutic target to prevent ALL dissemination [191]. Another study suggested the potential role of myosin subtypes in promoting leukaemia cell mobilisation, either in T‐ or B‐cell‐derived cell lines; thereby highlighting the therapeutic potential of this class of molecules in interfering with leukaemic cells' migratory capacity [192]. Recently, another study on ALL patients correlated the expression levels of MYO1G—a class I myosin involved in membrane tension and regulation of cell velocity—with patients' diagnosis and treatment outcomes. In particular, the researchers observed increased expression in patients with specific genomic alterations and those who relapsed after treatment. This suggests that MYO1G might contribute to ALL development and the clinical traits of the patients, potentially relevant for improving patient stratification at diagnosis [193].

## Conclusions and perspectives

In this review, we have highlighted the fundamental role of mechanobiology in shaping B‐cell behaviour, from antigen sensing and activation to migration and survival, in both healthy and malignant contexts. Additionally, we have explored emerging technologies and experimental approaches that enable the study of cellular mechanics, providing new insights into B‐cell function and disease progression. However, it is important to note that several aspects of B‐cell mechanobiology remain underexplored. As noted, most studies on lymphocyte mechanobiology have not been conducted in B cells. This is likely due to technical limitations related to the fact that they grow in suspension in culture, their small size, plasticity and high velocity moving, which constrain the measurements and characterisation of their mechanical properties.

As a result, from a mechanotransduction perspective, the precise role of the BCR as a mechanosensory molecule remains ill‐defined, and data on other mechanosensory molecules, such as the cationic channels PIEZO, are limited. Considering that the BCR is the primary receptor involved in B‐cell activation and function, this gap of knowledge impairs the comprehension of B‐cell regulation, which might impact on B‐cell malignancy pathophysiology understanding and therapeutics' design. Regarding the cytoskeleton, more information is available, particularly concerning IS formation; however, most studies on interstitial leukocyte migration in a 3D environment, particularly the switching between different migration modes, have focused on other amoeboid‐migrating cells.

Now, these mechanisms should be validated in B cells for a better understanding of the mechanisms of B‐cell trafficking in both health and disease. In the nucleus, only a few studies have explored B‐cell NSK structure and lamin organisation. The same applies to nuclear shuttling proteins such as YAP1/TAZ, as well as the mechanical gauge role of the nucleus, which has been primarily investigated in T cells and dendritic cells. Given the high plasticity of B cells, we would expect the nucleus, the stiffest organelle in the cell, to rapidly rearrange its structure and organisation, allowing the cell to deform even in the presence of very tiny constrictions while maintaining the integrity of the nuclear content. Furthermore, the nuclear‐first configuration suggests that the nucleus acts as a navigator, guiding cells through body compartments. If these findings are confirmed for B cells, it would highlight a crucial knowledge gap regarding their nucleus, which will need to be filled for understanding the mechanisms of immune response and malignant B cells' dissemination.

The diverse technologies we have discussed could help overcome these limitations, paving the way for new discoveries in B‐cell mechanobiology, both in healthy and malignant states. Understanding the mechanobiological aspects of drug responsiveness in malignant B cells could open new therapeutic avenues.

Directly targeting mechanical properties, for instance, by using drugs that alter the cytoskeleton or disrupt integrin‐mediated signalling, may inhibit tumour growth and progression [170]. For instance, dasatinib, an inhibitor of the BCR‐ABL1 tyrosine kinase, is a therapy approved for the treatment of patients with chronic and advanced‐phase chronic myeloid leukaemia [194]. Also, defactinib, an inhibitor of FAK, may favour patients' outcomes in combination with other drugs [195]. Additionally, combining traditional chemotherapy with agents targeting mechanobiological pathways, particularly in patients with resistant tumours, may enhance drug effectiveness and delivery to target sites. For example, a recent study on different solid tumours linked the mechanical properties with the ECM of the tumours and the T‐cell infiltration capabilities *in vivo*. By inhibiting lysyl oxidase—an enzyme often upregulated in tumours and involved in collagen organisation—they mechanically modulated the ECM, improving T‐cell mobility within the tumour and delaying tumour progression [196, 197]. However, translating fundamental mechanobiological knowledge into clinical practice remains difficult. One of the primary challenges is the development of sophisticated experimental models that can accurately replicate tumour microenvironment mechanics. As we have highlighted, there is now a plethora of measurement techniques, imaging methods, and *in vitro* models available that allow for a personalised assessment of tumour biomechanics. We expect that in the coming years, studies will adapt their application to different settings, including the possibility to integrate the available measurement techniques with *in vitro* systems to characterise tumour mechanics in a more sophisticated manner, while reproducing the relevant microenvironmental settings. A second major challenge is the need to develop highly selective therapeutic approaches while carefully assessing associated side‐effect risks. Specifically, the transition from bench to bedside requires a detailed understanding of the mechanical deregulation underlying tumour initiation, to comprehend how tumours can modulate their mechanics and which are the players involved. This is particularly important because alterations in the physical microenvironment may also disrupt the mechanobiology of healthy cells, potentially leading to unintended adverse effects [198].

In summary, the growing field of mechanobiology holds promise for the development of tailored therapies, ultimately improving patient management and treatment outcomes.

We are optimistic that future advancements in B‐cell mechanobiology will provide valuable insights into a better understanding of B cells trafficking and adaptation mechanisms, particularly in the context of haematological cancers. Such discoveries could unveil new opportunities to identify and target new molecular players. However, to fully realise this progress, the development of new protocols and approaches tailored for nonadherent cells, such as B cells, will be essential.

We emphasise the critical need for further investigation into B‐cell mechanobiology, which we believe holds significant potential for advancing our understanding of B‐cell‐related haematological diseases.

## Author contributions

MS, MC and CS wrote and revised the manuscript.

## References

[1] Lebien TW and Tedder TF (2008) B lymphocytes: how they develop and function. Blood 112, 1570–1580. 18725575 10.1182/blood-2008-02-078071 PMC2518873

[2] Burrows PD and Cooper MD (1997) B cell development and differentiation. Curr Opin Immunol 9, 239–244. 9099791 10.1016/s0952-7915(97)80142-2

[3] Alberts B , Johnson A , Lewis J , Raff M , Roberts K and Walter P (2016) Lymphocytes and the cellular basis of adaptive immunity. In Molecular Biology of the Cell. Garland Science, New York, NY.

[4] Miyasaka M , Hata E , Tohya K and Hayasaka H (2016) Lymphocyte recirculation. In Encyclopedia of Immunobiology ( Ratcliffe MJH , ed.), pp. 486–492. Academic Press, Oxford.

[5] Shaheen S , Wan Z , Haneef K , Zeng Y , Jing W and Liu W (2019) B Cell Mechanosensing: A Mechanistic Overview. 1st edn. Elsevier Inc., Amsterdam, The Netherlands. 10.1016/bs.ai.2019.08.003 31699219

[6] Vogel V (2018) Unraveling the mechanobiology of extracellular matrix. Annu Rev Physiol 80, 353–387. 29433414 10.1146/annurev-physiol-021317-121312

[7] Du H , Winer DA and Liu WF (2022) Tuning immunity through tissue mechanotransduction. Nat Rev Immunol 23, 174–188. 35974148 10.1038/s41577-022-00761-w PMC9379893

[8] Martino F , Perestrelo AR , Vinarský V , Pagliari S and Forte G (2018) Cellular mechanotransduction: from tension to function. Front Physiol 9, 1–21. 30026699 10.3389/fphys.2018.00824 PMC6041413

[9] Bartolo V Di , Valitutti S , Michael F , Dustin L , Hivroz C and Saitakis M (2016) Biophysical aspects of T lymphocyte activation at the immune synapse. Front Immunol 7, 46. 26913033 10.3389/fimmu.2016.00046 PMC4753286

[10] Caillier A , Oleksyn D , Fowell DJ , Miller J and Oakes PW (2023) T cells use focal adhesions to pull themselves through confined environments. *bioRxiv* 223, 2023.10.16.562587. 10.1101/2023.10.16.562587 PMC11187980 38889096

[11] Sampietro M , Cassina V , Salerno D , Barbaglio F , Buglione E , Marrano CA , Campanile R , Scarfò L , Biedenweg D , Fregin B *et al*. (2023) The nanomechanical properties of CLL cells are linked to the actin cytoskeleton and are a potential target of BTK inhibitors. HemaSphere 7, e931. 37492437 10.1097/HS9.0000000000000931 PMC10365208

[12] Ameen Al‐Aghbar M , Jainarayanan AK , Dustin ML and Roffler SR (2022) The interplay between membrane topology and mechanical forces in regulating T cell receptor activity. Commun Biol 5, 40. 35017678 10.1038/s42003-021-02995-1 PMC8752658

[13] Alonso JL and Goldmann WH (2016) Cellular mechanotransduction. AIMS Biophys 3, 50–62.

[14] Panciera T , Azzolin L , Cordenonsi M and Piccolo S (2017) Mechanobiology of YAP and TAZ in physiology and disease. Nat Rev Mol Cell Biol 18, 758–770. 28951564 10.1038/nrm.2017.87 PMC6192510

[15] Solis AG , Bielecki P , Steach HR , Sharma L , Harman CCD , Yun S , de Zoete MR , Warnock JN , To SDF , York AG *et al*. (2019) Mechanosensation of cyclical force by PIEZO1 is essential for innate immunity. Nature 573, 69–74. 31435009 10.1038/s41586-019-1485-8 PMC6939392

[16] Klymenko T , Bloehdorn J , Bahlo J , Robrecht S , Akylzhanova G , Cox K , Estenfelder S , Wang J , Edelmann J , Strefford JC *et al*. (2018) Lamin B1 regulates somatic mutations and progression of B‐cell malignancies. Leukemia 32, 364–375. 28804121 10.1038/leu.2017.255 PMC5808072

[17] Sugimura K , Lenne P‐F and Graner F (2016) Measuring forces and stresses in situ in living tissues. Development 143, 186–196. 26786209 10.1242/dev.119776

[18] Shin HY , Fukuda S and Schmid‐Schönbein GW (2021) Fluid shear stress‐mediated mechanotransduction in circulating leukocytes and its defect in microvascular dysfunction. J Biomech 120, 110394. 33784517 10.1016/j.jbiomech.2021.110394

[19] Apoorva F , Loiben AM , Shah SB , Purwada A , Fontan L , Goldstein R , Kirby BJ , Melnick AM , Cosgrove BD and Singh A (2018) How biophysical forces regulate human B cell lymphomas. Cell Rep 23, 499–511. 29642007 10.1016/j.celrep.2018.03.069 PMC5965297

[20] Upadhyaya A (2017) Mechanosensing in the immune response. Semin Cell Dev Biol 71, 137–145. 28830744 10.1016/j.semcdb.2017.08.031 PMC5747250

[21] Chatterjee S (2018) Endothelial mechanotransduction, redox signaling and the regulation of vascular inflammatory pathways. Front Physiol 9, 524. 29930512 10.3389/fphys.2018.00524 PMC5999754

[22] Jafarnejad M , Woodruff MC , Zawieja DC , Carroll MC and Moore JE (2015) Modeling lymph flow and fluid exchange with blood vessels in lymph nodes. Lymphat Res Biol 13, 234–247. 26683026 10.1089/lrb.2015.0028 PMC4685511

[23] Janmey PA , Fletcher DA and Reinhart‐King CA (2020) Stiffness sensing by cells. Physiol Rev 100, 695–724. 31751165 10.1152/physrev.00013.2019 PMC7276923

[24] Murrell M , Oakes PW , Lenz M and Gardel ML (2015) Forcing cells into shape: the mechanics of actomyosin contractility. Nat Rev Mol Cell Biol 16, 486–498. 26130009 10.1038/nrm4012 PMC7443980

[25] Yuseff MI , Pierobon P , Reversat A and Lennon‐Duménil AM (2013) How B cells capture, process and present antigens: a crucial role for cell polarity. Nat Rev Immunol 137, 475–486. 10.1038/nri3469 23797063

[26] Zhang T , Hu W and Chen W (2021) Plasma membrane integrates biophysical and biochemical regulation to trigger immune receptor functions. Front Immunol 12, 613185. 33679752 10.3389/fimmu.2021.613185 PMC7933204

[27] Sezgin E , Levental I , Mayor S and CE (2017) The mystery of membrane organization: composition, regulation and physiological relevance of lipid rafts. Nat Rev Mol Cell Biol 18, 361–374. 28356571 10.1038/nrm.2017.16 PMC5500228

[28] Le Roux AL , Quiroga X , Walani N , Arroyo M and Roca‐Cusachs P (2019) The plasma membrane as a mechanochemical transducer. Philos Trans R Soc B Biol Sci 374, 20180221. 10.1098/rstb.2018.0221 PMC6627014 31431176

[29] Karnell FG , Brezski RJ , King LB , Silverman MA and Monroe JG (2005) Membrane cholesterol content accounts for developmental differences in surface B cell receptor compartmentalization and signaling. J Biol Chem 280, 25621–25628. 15878848 10.1074/jbc.M503162200

[30] Elliott JI , Surprenant A , Marelli‐Berg FM , Cooper JC , Cassady‐Cain RL , Wooding C , Linton K , Alexander DR and Higgins CF (2005) Membrane phosphatidylserine distribution as a non‐apoptotic signalling mechanism in lymphocytes. Nat Cell Biol 7, 808–816. 16025105 10.1038/ncb1279

[31] Zhu C , Chen W , Lou J , Rittase W and Li K (2019) Mechanosensing through immunoreceptors. Nat Immunol 20, 1269–1278. 31534240 10.1038/s41590-019-0491-1 PMC7592628

[32] Ramesh S , Park S , Im W , Call MJ and Call ME (2022) T cell and B cell antigen receptors share a conserved core transmembrane structure. Proc Natl Acad Sci USA 119, e2208058119. 36409917 10.1073/pnas.2208058119 PMC9860311

[33] Judokusumo E , Tabdanov E , Kumari S , Dustin ML and Kam LC (2012) Mechanosensing in T lymphocyte activation. Biophys J 102, L5–L7. 22339876 10.1016/j.bpj.2011.12.011 PMC3260692

[34] Li Y‐C , Chen B‐M , Wu P‐C , Cheng T‐L , Kao L‐S , Tao M‐H , Lieber A and Roffler SR (2010) Cutting edge: mechanical forces acting on T cells immobilized via the TCR complex can trigger TCR signaling. J Immunol 184, 5959–5963. 20435924 10.4049/jimmunol.0900775

[35] Blumenthal D , Chandra V , Avery L and Burkhardt JK (2020) Mouse T cell priming is enhanced by maturation‐dependent stiffening of the dendritic cell cortex. elife 9, 1–44. 10.7554/eLife.55995 PMC7417170 32720892

[36] Wan Z , Zhang S , Fan Y , Liu K , Du F , Davey AM , Zhang H , Han W , Xiong C and Liu W (2013) B cell activation is regulated by the stiffness properties of the substrate presenting the antigens. J Immunol 190, 4661–4675. 23554309 10.4049/jimmunol.1202976

[37] Wan Z , Chen X , Chen H , Ji Q , Chen Y , Wang J , Cao Y , Wang F , Lou J , Tang Z *et al*. (2015) The activation of IgM‐ or isotype‐switched IgG‐ and IgE‐BCR exhibits distinct mechanical force sensitivity and threshold. elife 4, e06925. 26258882 10.7554/eLife.06925 PMC4555871

[38] Zeng Y , Yi J , Wan Z , Liu K , Song P , Chau A , Wang F , Chang Z , Han W , Zheng W *et al*. (2015) Substrate stiffness regulates B‐cell activation, proliferation, class switch, and T‐cell‐independent antibody responses in vivo. Eur J Immunol 45, 1621–1634. 25756957 10.1002/eji.201444777

[39] Wang J , Lin F , Wan Z , Sun X , Lu Y , Huang J , Wang F , Zeng Y , Chen Y‐H , Shi Y *et al*. (2018) Profiling the origin, dynamics, and function of traction force in B cell activation. Sci Signal 11, eaai9192. 30087179 10.1126/scisignal.aai9192

[40] Hynes RO (2002) Integrins: bidirectional, allosteric signaling machines. Cell 110, 673–687. 12297042 10.1016/s0092-8674(02)00971-6

[41] Arana E , Harwood NE and Batista FD (2008) Regulation of integrin activation through the B‐cell receptor. J Cell Sci 121, 2279–2286. 18596256 10.1242/jcs.017905

[42] Burridge K (2017) Focal adhesions: a personal perspective on a half century of progress. FEBS J 284, 3355–3361. 28796323 10.1111/febs.14195 PMC5643231

[43] Stutchbury B , Atherton P , Tsang R , Wang DY and Ballestrem C (2017) Distinct focal adhesion protein modules control different aspects of mechanotransduction. J Cell Sci 130, 1612–1624. 28302906 10.1242/jcs.195362 PMC5450230

[44] Lachowski D , Cortes E , Robinson B , Rice A , Rombouts K and Del Río Hernández AE (2018) FAK controls the mechanical activation of YAP, a transcriptional regulator required for durotaxis. FASEB J 32, 1099–1107. 29070586 10.1096/fj.201700721R

[45] Shaheen S , Wan Z , Li Z , Chau A , Li X , Zhang S , Liu Y , Yi J , Zeng Y , Wang J *et al*. (2017) Substrate stiffness governs the initiation of b cell activation by the concerted signaling of PKCβ and focal adhesion kinase. elife 6, 1–29. 10.7554/eLife.23060 PMC5536945 28755662

[46] Coste B , Mathur J , Schmidt M , Earley TJ , Ranade S , Petrus MJ , Dubin AE and Patapoutian A (2010) Piezo1 and Piezo2 are essential components of distinct mechanically‐activated cation channels. Science 330, 55–60. 20813920 10.1126/science.1193270 PMC3062430

[47] Kefauver JM , Ward AB and Patapoutian A (2020) Discoveries in structure and physiology of mechanically activated ion channels. Nature 587, 567–576. 33239794 10.1038/s41586-020-2933-1 PMC8477435

[48] Zhang Y , Zou W , Dou W , Luo H and Ouyang X (2024) Pleiotropic physiological functions of Piezo1 in human body and its effect on malignant behavior of tumors. Front Physiol 15, 1–20. 10.3389/fphys.2024.1377329 PMC11058998 38690080

[49] Wang Y , Yang H , Jia A , Wang Y , Yang Q , Dong Y , Hou Y , Cao Y , Dong L , Bi Y *et al*. (2022) Dendritic cell Piezo1 directs the differentiation of TH1 and Treg cells in cancer. elife 11, e79957. 35993548 10.7554/eLife.79957 PMC9451538

[50] Liu CSC , Raychaudhuri D , Paul B , Chakrabarty Y , Ghosh AR , Rahaman O , Talukdar A and Ganguly D (2018) Cutting edge: Piezo1 Mechanosensors optimize human T cell activation. J Immunol 200, 1255–1260. 29330322 10.4049/jimmunol.1701118

[51] Kwak K , Sohn H , George R , Torgbor C , Manzella‐Lapeira J , Brzostowski J and Pierce SK (2023) B cell responses to membrane‐presented antigens require the function of the mechanosensitive cation channel Piezo1. Sci Signal 16, eabq5096. 37751477 10.1126/scisignal.abq5096 PMC10691204

[52] Wang JC , Yim Y‐I , Wu X , Jaumouille V , Cameron A , Waterman CM , Kehrl JH and Hammer JA (2022) A B‐cell actomyosin arc network couples integrin co‐stimulation to mechanical force‐dependent immune synapse formation. elife 11, e72805. 35404237 10.7554/eLife.72805 PMC9142150

[53] Kameritsch P and Renkawitz J (2020) Principles of leukocyte migration strategies. Trends Cell Biol 30, 818–832. 32690238 10.1016/j.tcb.2020.06.007

[54] Čada Š , Vondálová Blanářová O , Gömoryová K , Mikulová A , Bačovská P , Zezula N , Kumari Jadaun A , Janovská P , Plešingerová H and Bryja V (2022) Role of casein kinase 1 in the amoeboid migration of B‐cell leukemic and lymphoma cells: a quantitative live imaging in the confined environment. Front Cell Dev Biol 10, 911966. 36561363 10.3389/fcell.2022.911966 PMC9763939

[55] Lämmermann T , Bader BL , Monkley SJ , Worbs T , Wedlich‐Söldner R , Hirsch K , Keller M , Förster R , Critchley DR , Fässler R *et al*. (2008) Rapid leukocyte migration by integrin‐independent flowing and squeezing. Nature 453, 51–56. 18451854 10.1038/nature06887

[56] Reversat A , Gaertner F , Merrin J , Stopp J , Tasciyan S , Aguilera J , de Vries I , Hauschild R , Hons M , Piel M *et al*. (2020) Cellular locomotion using environmental topography. Nature 582, 582–585. 32581372 10.1038/s41586-020-2283-z

[57] Renkawitz J , Schumann K , Weber M , Lämmermann T , Pflicke H , Piel M , Polleux J , Spatz JP and Sixt M (2009) Adaptive force transmission in amoeboid cell migration. Nat Cell Biol 1112, 1438–1443. 10.1038/ncb1992 19915557

[58] Katakai T , Habiro K and Kinashi T (2013) Dendritic cells regulate high‐speed interstitial T cell migration shapein the lymph node via LFA‐1/ICAM‐1. J Immunol 191, 1188–1199. 23817428 10.4049/jimmunol.1300739

[59] Hons M , Kopf A , Hauschild R , Leithner A , Gaertner F , Abe J , Renkawitz J , Stein JV and Sixt M (2018) Chemokines and integrins independently tune actin flow and substrate friction during intranodal migration of T cells. Nat Immunol 19, 606–616. 29777221 10.1038/s41590-018-0109-z

[60] Van Helvert S , Storm C and Friedl P (2018) Mechanoreciprocity in cell migration. Nat Cell Biol 20, 8–20. 29269951 10.1038/s41556-017-0012-0 PMC5943039

[61] Bhanja A , Rey‐Suarez I , Song W and Upadhyaya A (2022) Bidirectional feedback between BCR signaling and actin cytoskeletal dynamics. FEBS J 289, 4430–4446. 34124846 10.1111/febs.16074 PMC8669062

[62] Lee J , Sengupta P , Brzostowski J , Lippincott‐Schwartz J and Pierce SK (2017) The nanoscale spatial organization of B‐cell receptors on immunoglobulin M– and G–expressing human B‐cells. Mol Biol Cell 28, 511–523. 27974642 10.1091/mbc.E16-06-0452 PMC5305258

[63] Tolar P (2017) Cytoskeletal control of B cell responses to antigens. Nat Rev Immunol 17, 621–634. 28690317 10.1038/nri.2017.67

[64] Liu C , Miller H , Orlowski G , Hang H , Upadhyaya A and WS (2012) Actin reorganization is required for the formation of polarized B cell receptor signalosomes in response to both soluble and membrane‐ associated antigens. J Immunol 188, 3237–3246. 22387556 10.4049/jimmunol.1103065 PMC3312033

[65] Fleire SJ , Goldman JP , Carrasco YR , Weber M , Bray D and Batista FD (2006) B cell ligand discrimination through a spreading and contraction response. Science 312, 738–741. 16675699 10.1126/science.1123940

[66] Liu C , Bai X , Wu J , Sharma S and Upadhyaya A (2013) N‐WASP is essential for the negative regulation of B cell receptor signaling. PLoS Biol 11, 1001704. 10.1371/journal.pbio.1001704 PMC3818172 24223520

[67] Spillane KM and Tolar P (2018) DNA‐based probes for measuring mechanical forces in cell‐cell contacts: application to B cell antigen extraction from immune synapses. Methods Mol Biol 1707, 69–80. 29388100 10.1007/978-1-4939-7474-0_5

[68] McShane AN and Malinova D (2022) The ins and outs of antigen uptake in B cells. Front Immunol 13, 892169. 35572544 10.3389/fimmu.2022.892169 PMC9097226

[69] Boulant S , Kural C , Zeeh JC , Ubelmann F and Kirchhausen T (2011) Actin dynamics counteract membrane tension during clathrin‐mediated endocytosis. Nat Cell Biol 13, 1124–1132. 21841790 10.1038/ncb2307 PMC3167020

[70] Stoddart A , Jackson AP and Brodsky FM (2005) Plasticity of B cell receptor internalization upon conditional depletion of Clathrin. Mol Biol Cell 16, 2339–2348. 15716350 10.1091/mbc.E05-01-0025 PMC1087239

[71] Roberts AD , Davenport TM , Dickey AM , Ahn R , Sochacki KA and Taraska JW (2020) Structurally distinct endocytic pathways for B cell receptors in B lymphocytes. Mol Biol Cell 31, 2826–2840. 33085561 10.1091/mbc.E20-08-0532 PMC7851864

[72] Kirby TJ and Lammerding J (2018) Emerging views of the nucleus as a cellular mechanosensor. Nat Cell Biol 20, 373–381. 29467443 10.1038/s41556-018-0038-y PMC6440800

[73] Venturini V , Pezzano F , Castro FC , Häkkinen HM , Jiménez‐Delgado S , Colomer‐Rosell M , Marro M , Tolosa‐Ramon Q , Paz‐López S , Valverde MA *et al*. (2020) The nucleus measures shape changes for cellular proprioception to control dynamic cell behavior. Science 370, eaba2644. 33060331 10.1126/science.aba2644

[74] Lomakin AJ , Cattin CJ , Cuvelier D , Alraies Z , Molina M , Nader GPF , Srivastava N , Saez PJ , Garcia‐Arcos JM , Zhitnyak IY *et al*. (2020) The nucleus acts as a ruler tailoring cell responses to spatial constraints. Science 370, eaba2894. 33060332 10.1126/science.aba2894 PMC8059074

[75] Renkawitz J , Kopf A , Stopp J , de Vries I , Driscoll MK , Merrin J , Hauschild R , Welf ES , Danuser G , Fiolka R *et al*. (2019) Nuclear positioning facilitates amoeboid migration along the path of least resistance. Nature 568, 546–550. 30944468 10.1038/s41586-019-1087-5 PMC7217284

[76] Valbuena A , Vera AM , Oroz J , Menéndez M and Carrión‐Vázquez M (2012) Mechanical properties of β‐catenin revealed by single‐molecule experiments. Biophys J 103, 1744–1752. 23083718 10.1016/j.bpj.2012.07.051 PMC3475332

[77] Wang Y , Wang D and Guo J (2019) Zyxin: a mechanotransductor to regulate gene expression. Eur Rev Med Pharmacol Sci 23, 413–425. 30657586 10.26355/eurrev_201901_16790

[78] Sathe AR , Shivashankar GV and Sheetz MP (2016) Nuclear transport of paxillin depends on focal adhesion dynamics and FAT domains. J Cell Sci 129, 1981–1988. 27068537 10.1242/jcs.172643 PMC4895192

[79] Dupont S , Morsut L , Aragona M , Enzo E , Giulitti S , Cordenonsi M , Zanconato F , Le Digabel J , Forcato M , Bicciato S *et al*. (2011) Role of YAP/TAZ in mechanotransduction. Nature 474, 179–183. 21654799 10.1038/nature10137

[80] van Sciver N , Ohashi M , Pauly NP , Bristol JA , Nelson SE , Johannsen EC and Kenney SC (2021) Hippo signaling effectors YAP and TAZ induce Epstein‐Barr virus (EBV) lytic reactivation through TEADs in epithelial cells. PLoS Pathog 17, e1009783. 34339458 10.1371/journal.ppat.1009783 PMC8360610

[81] Pan Z , Tian Y , Cao C and Niu G (2019) The emerging role of YAP/TAZ in tumor immunity. Mol Cancer Res 17, 1777–1786. 31308148 10.1158/1541-7786.MCR-19-0375

[82] Bai X , Huang L , Niu L , Zhang Y , Wang J , Sun X , Jiang H , Zhang Z , Miller H , Tao W *et al*. (2016) Mst1 positively regulates B‐cell receptor signaling via CD19 transcriptional levels. Blood Adv 1, 219–230. 29296937 10.1182/bloodadvances.2016000588 PMC5737167

[83] Perez‐Lopez A , Rosales‐Reyes R , Alpuche‐Aranda CM and VO‐N (2013) Salmonella downregulates nod‐like receptor family CARD domain containing protein 4 expression to promote its survival in B cells by preventing inflammasome activation and cell death. J Immunol 190, 1201–1209. 23284055 10.4049/jimmunol.1200415

[84] Janota CS , Calero‐Cuenca FJ and Gomes ER (2020) The role of the cell nucleus in mechanotransduction. Curr Opin Cell Biol 63, 204–211. 32361559 10.1016/j.ceb.2020.03.001

[85] Adam SA (2017) The nucleoskeleton. Cold Spring Harb Perspect Biol 9, 79–80. 10.1101/cshperspect.a023556 PMC5287075 28148597

[86] Ulloa R , Corrales O , Cabrera‐Reyes F , Jara‐Wilde J , Saez JJ , Rivas C , Lagos J , Härtel S , Quiroga C , Yuseff MI *et al*. (2022) B cells adapt their nuclear morphology to organize the immune synapse and facilitate antigen extraction. Front Immunol 12, 1–16. 10.3389/fimmu.2021.801164 PMC8863768 35222354

[87] Guilluy C , Osborne LD , Van Landeghem L , Sharek L , Superfine R , Garcia‐Mata R and Burridge K (2014) Isolated nuclei adapt to force and reveal a mechanotransduction pathway in the nucleus. Nat Cell Biol 16, 376–381. 24609268 10.1038/ncb2927 PMC4085695

[88] Sullivan T , Escalante‐Alcalde D , Bhatt H , Anver M , Bhat N , Nagashima K , Stewart CL and Burke B (1999) Loss of A‐type lamin expression compromises nuclear envelope integrity leading to muscular dystrophy. J Cell Biol 147, 913–919. 10579712 10.1083/jcb.147.5.913 PMC2169344

[89] Lammerding J , Fong LG , Ji JY , Reue K , Stewart CL , Young SG and Lee RT (2006) Lamins a and C but not lamin B1 regulate nuclear mechanics. J Biol Chem 281, 25768–25780. 16825190 10.1074/jbc.M513511200

[90] Hale JS , Frock RL , Mamman SA , Fink PJ and Kennedy BK (2010) Cell‐extrinsic defective lymphocyte development in Lmna(−/−) mice. PLoS One 5, e10127. 20405040 10.1371/journal.pone.0010127 PMC2853576

[91] Vahabikashi A , Sivagurunathan S , Ann Sadsad Nicdao F , Long Han Y , Young Park C , Kittisopikul M , Wong X , Tran JR , Gundersen GG , Reddy KL *et al*. (2022) Nuclear lamin isoforms differentially contribute to LINC complex‐dependent nucleocytoskeletal coupling and whole‐cell mechanics. Proc Natl Acad Sci USA 119, e2121816119. 35439057 10.1073/pnas.2121816119 PMC9170021

[92] Swift J , Ivanovska IL , Buxboim A , Harada T , Dave PCP , Pinter J , Pajerowski JD , Spinler KR , Shin J , Rehfeldt F *et al*. (2014) Nuclear Lamin‐a scales with tissue stiffness and enhances matrix‐directed differentiation. Science 341, 1–33. 10.1126/science.1240104 PMC3976548 23990565

[93] Wang M , Ivanovska I , Vashisth M and Discher DE (2022) Nuclear mechanoprotection: from tissue atlases as blueprints to distinctive regulation of nuclear lamins. APL Bioeng 6, 021504. 35719698 10.1063/5.0080392 PMC9203124

[94] Shin JW , Spinler KR , Swift J , Chasis JA , Mohandas N and Discher DE (2013) Lamins regulate cell trafficking and lineage maturation of adult human hematopoietic cells. Proc Natl Acad Sci USA 110, 18892–18897. 24191023 10.1073/pnas.1304996110 PMC3839750

[95] Rowat AC , Jaalouk DE , Zwerger M , Ung WL , Eydelnant IA , Olins DE , Olins AL , Herrmann H , Weitz DA and Lammerding J (2013) Nuclear envelope composition determines the ability of neutrophil‐type cells to passage through micron‐scale constrictions. J Biol Chem 288, 8610–8618. 23355469 10.1074/jbc.M112.441535 PMC3605679

[96] Saez A , Herrero‐Fernandez B , Gomez‐Bris R , Somovilla‐Crespo B , Rius C and Gonzalez‐Granado JM (2020) Lamin A/C and the immune system: one intermediate filament, Many Faces. Int J Mol Sci 21, 6109. 32854281 10.3390/ijms21176109 PMC7504305

[97] González‐Granado JM , Silvestre‐Roig C , Rocha‐Perugini V , Trigueros‐Motos L , Cibrián D , Morlino G , Blanco‐Berrocal M , Osorio FG , Freije JMP , López‐Otín C *et al*. (2014) Nuclear envelope lamin‐A couples actin dynamics with immunological synapse architecture and T cell activation. Sci Signal 7, ra37. 24757177 10.1126/scisignal.2004872 PMC4337980

[98] Sherif M , Schäfer H , Scharf S , van Oostendorp V , Sadeghi Shoreh Deli A , Loth AG , Piel M , Hansmann ML , Oellerich T , Fend F *et al*. (2024) EZB‐type diffuse large B‐cell lymphoma cell lines have superior migration capabilities compared to MCD‐type. Br J Haematol 205, 2327–2337. 39355919 10.1111/bjh.19778 PMC11637725

[99] González‐Bermúdez B , Kobayashi H , Abarca‐Ortega A , Córcoles‐Lucas M , González‐Sánchez M , De la Fuente M , Guinea GV , Elices M and Plaza GR (2022) Aging is accompanied by T‐cell stiffening and reduced interstitial migration through dysfunctional nuclear organization. Immunology 167, 622–639. 36054660 10.1111/imm.13559

[100] Samassa F , Ferrari ML , Husson J , Mikhailova A , Porat Z , Sidaner F , Brunner K , Teo TH , Frigimelica E , Tinevez JY *et al*. (2020) Shigella impairs human T lymphocyte responsiveness by hijacking actin cytoskeleton dynamics and T cell receptor vesicular trafficking. Cell Microbiol 22, e13166. 31957253 10.1111/cmi.13166 PMC7187243

[101] Swaminathan V , Mythreye K , O'Brien TE , Berchuck A , Blobe GC and Superfine R (2011) Mechanical stiffness grades metastatic potential in patient tumor cells and in cancer cell lines. Cancer Res 71, 5075. 21642375 10.1158/0008-5472.CAN-11-0247 PMC3220953

[102] Thomas G , Burnham NA , Camesano TA and Wen Q (2013) Measuring the mechanical properties of living cells using atomic force microscopy. J Vis Exp 50497. doi: 10.3791/50497 23851674 PMC3729185

[103] Sokolov I , Dokukin ME and Guz NV (2013) Method for quantitative measurements of the elastic modulus of biological cells in AFM indentation experiments. Methods 60, 202–213. 23639869 10.1016/j.ymeth.2013.03.037

[104] Li M , Liu L , Xiao X , Xi N and Wang Y (2016) Viscoelastic properties measurement of human lymphocytes by atomic force microscopy based on magnetic beads cell isolation. IEEE Trans Nanobioscience 15, 398–411. 28113818 10.1109/TNB.2016.2547639

[105] Guilak F , Tedrow JR and Burgkart R (2000) Viscoelastic properties of the cell nucleus. Biochem Biophys Res Commun 269, 781–786. 10720492 10.1006/bbrc.2000.2360

[106] Jalili N and Laxminarayana K (2004) A review of atomic force microscopy imaging systems: application to molecular metrology and biological sciences. Mechatronics 14, 907–945.

[107] Noy A (2011) Force spectroscopy 101: how to design, perform, and analyze an AFM‐based single molecule force spectroscopy experiment. Curr Opin Chem Biol 15, 710–718. 21862386 10.1016/j.cbpa.2011.07.020

[108] Li M , Liu L , Xi N , Wang Y , Dong Z , Tabata O , Xiao X and Zhang W (2011) Imaging and measuring the rituximab‐induced changes of mechanical properties in B‐lymphoma cells using atomic force microscopy. Biochem Biophys Res Commun 404, 689–694. 21156157 10.1016/j.bbrc.2010.12.043

[109] Campanile R , Helenius J , Scielzo C , Scarfò L , Salerno D , Bossi M , Falappi M , Saponara A , Müller DJ , Mantegazza F *et al*. (2025) Production of AFM wedged cantilevers for stress‐relaxation experiments: uniaxial loading of soft, spherical cells. Methods 236, 1–9. 39971021 10.1016/j.ymeth.2025.02.004

[110] Hao Y , Cheng S , Tanaka Y , Hosokawa Y , Yalikun Y and Li M (2020) Mechanical properties of single cells: measurement methods and applications. Biotechnol Adv 45, 107648. 33080313 10.1016/j.biotechadv.2020.107648

[111] Bufi N , Saitakis M , Dogniaux S , Buschinger O , Bohineust A , Richert A , Maurin M , Hivroz C and Asnacios A (2015) Human primary immune cells exhibit distinct mechanical properties that are modified by inflammation. Biophys J 108, 2181–2190. 25954876 10.1016/j.bpj.2015.03.047 PMC4423053

[112] Mustapha F , Sengupta K and Puech PH (2022) May the force be with your (immune) cells: an introduction to traction force microscopy in immunology. Front Immunol 13, 898558. 35990636 10.3389/fimmu.2022.898558 PMC9389945

[113] Schoen I , Hu W , Klotzsch E and Vogel V (2010) Probing cellular traction forces by micropillar arrays: contribution of substrate warping to pillar deflection. Nano Lett 10, 1823–1830. 20387859 10.1021/nl100533c PMC2881340

[114] Kumari A , Pineau J , Lennon‐Duménil AM , Balland M and Pierobon P (2020) Traction force microscopy to study b lymphocyte activation. J Vis Exp 1–15. doi: 10.3791/60947 32773764

[115] Kumari A , Pineau J , Sáez PJ , Maurin M , Lankar D , San Roman M , Hennig K , Boura VF , Voituriez R , Karlsson MCI *et al*. (2019) Actomyosin‐driven force patterning controls endocytosis at the immune synapse. Nat Commun 10, 2870. 31253773 10.1038/s41467-019-10751-7 PMC6599028

[116] Catala‐Castro F , Schäffer E and Krieg M (2022) Exploring cell and tissue mechanics with optical tweezers. J Cell Sci 135, jcs259355. 35942913 10.1242/jcs.259355

[117] Verdeny I , Farré A , Mas J , López‐Quesada C , Martín‐Badosa E and Montes‐Usategui M (2011) Optical trapping: a review of essential concepts. Opt Pura Apl 44, 527–551.

[118] Ashkin A (1970) Acceleration and trapping of particles by radiation pressure. Phys Rev Lett 24, 156–159.

[119] Guck J , Ananthakrishnan R , Mahmood H , Moon TJ , Cunningham CC and Käs J (2001) The optical stretcher: a novel laser tool to micromanipulate cells. Biophys J 81, 767–784. 11463624 10.1016/S0006-3495(01)75740-2 PMC1301552

[120] Chan CJ , Whyte G , Boyde L , Salbreux G and Guck J (2014) Impact of heating on passive and active biomechanics of suspended cells. Interface Focus 4, 20130069. 24748957 10.1098/rsfs.2013.0069 PMC3982451

[121] Roth KB , Neeves KB , Squier J and Marr DWM (2016) High‐throughput linear optical stretcher for mechanical characterization of blood cells. Cytom Part A 89, 391–397. 10.1002/cyto.a.22794 PMC10625799 26565892

[122] Avsievich T , Zhu R , Popov A , Bykov A and Meglinski I (2020) The advancement of blood cell research by optical tweezers. Rev Phys 5, 100043.

[123] Yang T , Bragheri F , Nava G , Chiodi I , Mondello C , Osellame R , Berg‐Sørensen K , Cristiani I and Minzioni P (2016) A comprehensive strategy for the analysis of acoustic compressibility and optical deformability on single cells. Sci Rep 6, 1–13. 27040456 10.1038/srep23946 PMC4819226

[124] Ding X , Peng Z , Lin SCS , Geri M , Li S , Li P , Chen Y , Dao M , Suresh S and Huang TJ (2014) Cell separation using tilted‐angle standing surface acoustic waves. Proc Natl Acad Sci USA 111, 12992–12997. 25157150 10.1073/pnas.1413325111 PMC4246961

[125] Wu Y , Stewart AG and Lee PVS (2019) On‐chip cell mechanophenotyping using phase modulated surface acoustic wave. Biomicrofluidics 13, 024107. 31065306 10.1063/1.5084297 PMC6478592

[126] Kabakova I , Zhang J , Xiang Y , Caponi S , Bilenca A , Guck J and Scarcelli G (2024) Brillouin microscopy. Nat Rev Methods Prim 4, 8. 10.1038/s43586-023-00286-z PMC11465583 39391288

[127] Prevedel R , Diz‐Muñoz A , Ruocco G and Antonacci G (2019) Brillouin microscopy: an emerging tool for mechanobiology. Nat Methods 16, 969–977. 31548707 10.1038/s41592-019-0543-3

[128] Hochmuth RM (2000) Micropipette aspiration of living cells. J Biomech 33, 15–22. 10609514 10.1016/s0021-9290(99)00175-x

[129] Lee LM and Liu AP (2014) The application of micropipette aspiration in molecular mechanics of single cells. J Nanotechnol Eng Med 5, 1–6. 10.1115/1.4029936 PMC4476029 26155329

[130] González‐Bermúdez B , Guinea GV and Plaza GR (2019) Advances in micropipette aspiration: applications in cell biomechanics, models, and extended studies. Biophys J 116, 587–594. 30683304 10.1016/j.bpj.2019.01.004 PMC6383002

[131] Waugh RE , Lomakina E , Amitrano A and Kim M (2023) Activation effects on the physical characteristics of T lymphocytes. Front Bioeng Biotechnol 11, 1–11. 10.3389/fbioe.2023.1175570 PMC10225623 37256117

[132] Guillou L , Babataheri A , Saitakis M , Bohineust A , Dogniaux S , Hivroz C , Barakat AI and Husson J (2016) T‐lymphocyte passive deformation is controlled by unfolding of membrane surface reservoirs. Mol Biol Cell 27, 3574–3582. 27605708 10.1091/mbc.E16-06-0414 PMC5221589

[133] Wilson DI (2017) What is rheology? Eye 322, 179–183. 10.1038/eye.2017.267 PMC5811736 29271417

[134] Otto O , Rosendahl P , Mietke A , Golfier S , Herold C , Klaue D , Girardo S , Pagliara S , Ekpenyong A , Jacobi A *et al*. (2015) Real‐time deformability cytometry: on‐the‐fly cell mechanical phenotyping. Nat Methods 12, 199–202. 25643151 10.1038/nmeth.3281

[135] Mietke A , Otto O , Girardo S , Rosendahl P , Taubenberger A , Golfier S , Ulbricht E , Aland S , Guck J and Fischer‐Friedrich E (2015) Extracting cell stiffness from real‐time deformability cytometry: theory and experiment. Biophys J 109, 2023–2036. 26588562 10.1016/j.bpj.2015.09.006 PMC4656812

[136] Heragu S (2019) Real‐time deformability cytometry: label‐free functional characterization of cells. Handb Deterg Part D 1656, 229–251.

[137] Golfier S , Rosendahl P , Mietke A , Herbig M , Guck J and Otto O (2017) High‐throughput cell mechanical phenotyping for label‐free titration assays of cytoskeletal modifications. Cytoskeleton (Hoboken) 74, 283–296. 28445605 10.1002/cm.21369 PMC5601209

[138] Fregin B , Czerwinski F , Biedenweg D , Girardo S , Gross S , Aurich K and Otto O (2019) High‐throughput single‐cell rheology in complex samples by dynamic real‐time deformability cytometry. Nat Commun 10, 415. 30679420 10.1038/s41467-019-08370-3 PMC6346011

[139] Lavrenyuk K , Conway D and Dahl KN (2021) Imaging methods in mechanosensing: a historical perspective and visions for the future. Mol Biol Cell 32, 842–854. 33788578 10.1091/mbc.E20-10-0671 PMC8108522

[140] Seghir R and Arscott S (2015) Extended PDMS stiffness range for flexible systems. Sens Actuators A Phys 230, 33–39.

[141] Zeng Q , Xu B , Deng J , Shang K , Guo Z and Wu S (2024) Optimization of polydimethylsiloxane (PDMS) surface chemical modification and formulation for improved T cell activation and expansion. Colloids Surf B Biointerfaces 239, 113977. 38776594 10.1016/j.colsurfb.2024.113977

[142] Minaisah RM , Cox S and Warren DT (2016) The use of polyacrylamide hydrogels to study the effects of matrix stiffness on nuclear envelope properties. Methods Mol Biol 1411, 233–239. 27147046 10.1007/978-1-4939-3530-7_15

[143] Shakiba D , Genin GM and Zustiak SP (2023) Mechanobiology of cancer cell responsiveness to chemotherapy and immunotherapy: mechanistic insights and biomaterial platforms. Adv Drug Deliv Rev 196, 114771. 36889646 10.1016/j.addr.2023.114771 PMC10133187

[144] Jensen C and Teng Y (2020) Is it time to start transitioning from 2D to 3D cell culture? Front Mol Biosci 7, 1–15. 32211418 10.3389/fmolb.2020.00033 PMC7067892

[145] Baruffaldi D , Palmara G , Pirri C and Frascella F (2021) 3D cell culture: recent development in materials with tunable stiffness. ACS Appl Bio Mater 4, 2233–2250. 10.1021/acsabm.0c01472 35014348

[146] Du EY , Jung MS , Skhinas J , Tolentino MAK , Noy J , Jamshidi N , Houng JL , Tjandra KC , Engel M , Utama R *et al*. (2023) 3D bioprintable hydrogel with tunable stiffness for exploring cells encapsulated in matrices of differing stiffnesses. ACS Appl Bio Mater 6, 4603–4612. 10.1021/acsabm.3c00334 37844275

[147] Zhong Z , Quiñones‐Pérez M , Dai Z , Juarez VM , Bhatia E , Carlson CR , Shah SB , Patel A , Fang Z , Hu T *et al*. (2024) Human immune organoids to decode B cell response in healthy donors and patients with lymphoma. Nat Mater 242, 297–311. 10.1038/s41563-024-02037-1 PMC11866935 39506098

[148] Braham MVJ , van Binnendijk RS , Buisman AMM , Mebius RE , de Wit J and van Els CACM (2022) A synthetic human 3D in vitro lymphoid model enhancing B‐cell survival and functional differentiation. iScience 26, 105741. 36590159 10.1016/j.isci.2022.105741 PMC9794978

[149] Hoarau‐Véchot J , Rafii A , Touboul C and Pasquier J (2018) Halfway between 2D and animal models: are 3D cultures the ideal tool to study cancer‐microenvironment interactions? Int J Mol Sci 19, 181. 29346265 10.3390/ijms19010181 PMC5796130

[150] Polachecka WJ , Lib R , Uzela SGM and Kamm RD (2013) Microfluidic platforms for mechanobiology. Lab Chip 13, 2252–2267. 23649165 10.1039/c3lc41393d PMC3714214

[151] Urbanska M , Muñoz HE , Shaw Bagnall J , Otto O , Manalis SR , Di Carlo D and Guck J (2020) A comparison of microfluidic methods for high‐throughput cell deformability measurements. Nat Methods 17, 587–593. 32341544 10.1038/s41592-020-0818-8 PMC7275893

[152] Cook CA , Huri PY , Ginn BP , Gilbert‐Honick J , Somers SM , Temple JP , Mao HQ and Grayson WL (2016) Characterization of a novel bioreactor system for 3D cellular mechanobiology studies. Biotechnol Bioeng 113, 1825–1837. 26825810 10.1002/bit.25946

[153] Raveling AR , Theodossiou SK and Schiele NR (2018) A 3D printed mechanical bioreactor for investigating mechanobiology and soft tissue mechanics. MethodsX 5, 924–932. 30167382 10.1016/j.mex.2018.08.001 PMC6111048

[154] Liu Y , Ren X , Wu J , Wilkins JA and Lin F (2022) T cells chemotaxis migration studies with a multi‐channel microfluidic device. Micromachines 13, 1567. 36295920 10.3390/mi13101567 PMC9611841

[155] Van Der Meer AD , Poot AA , Feijen J and Vermes I (2010) Analyzing shear stress‐induced alignment of actin filaments in endothelial cells with a microfluidic assay. Biomicrofluidics 4, 1–5. 10.1063/1.3366720 PMC2905259 20644662

[156] Sundararaghavan HG , Monteiro GA , Firestein BL and Shreiber DI (2009) Neurite growth in 3D collagen gels with gradients of mechanical properties. Biotechnol Bioeng 102, 632–643. 18767187 10.1002/bit.22074

[157] Miller JS , Stevens KR , Yang MT , Baker BM , Nguyen DHT , Cohen DM , Toro E , Chen AA , Galie PA , Yu X *et al*. (2012) Rapid casting of patterned vascular networks for perfusable engineered three‐dimensional tissues. Nat Mater 119, 768–774. 10.1038/nmat3357 PMC3586565 22751181

[158] Le Berre M , Zlotek‐Zlotkiewicz E , Bonazzi D , Lautenschlaeger F and Piel M (2014) Methods for two‐dimensional cell confinement. Methods Cell Biol 121, 213–229. 24560512 10.1016/B978-0-12-800281-0.00014-2

[159] Sáez PJ , Barbier L , Attia R , Thiam HR , Piel M and Vargas P (2018) Leukocyte migration and deformation in collagen gels and microfabricated constrictions. Methods Mol Biol 1749, 361–373. 29526010 10.1007/978-1-4939-7701-7_26

[160] An L , Ji F , Zhao E , Liu Y and Liu Y (2023) Measuring cell deformation by microfluidics. Front Bioeng Biotechnol 11, 1–16. 10.3389/fbioe.2023.1214544 PMC10331473 37434754

[161] Thompson SB , Sandor AM , Lui V , Chung JW , Waldman MM , Long RA , Estin ML , Matsuda JL , Friedman RS and Jacobelli J (2020) Formin‐like 1 mediates effector t cell trafficking to inflammatory sites to enable t cell‐mediated autoimmunity. elife 9, 1–27. 10.7554/eLife.58046 PMC7308091 32510333

[162] McCormick S , Tong Z , Ivask A , Morozesk M , Voelcker NH , Lombi E and Priest C (2017) Optimization of binding B‐lymphocytes in a microfluidic channel: surface modification, stasis time and shear response. Biofabrication 10, 014101. 29058681 10.1088/1758-5090/aa9554

[163] Pattanayak P , Singh SK , Gulati M , Vishwas S , Kapoor B , Chellappan DK , Anand K , Gupta G , Jha NK , Gupta PK *et al*. (2021) Microfluidic chips: recent advances, critical strategies in design, applications and future perspectives. Microfluid Nanofluid 25, 99. 34720789 10.1007/s10404-021-02502-2 PMC8547131

[164] Le Berre M , Aubertin J and Piel M (2012) Fine control of nuclear confinement identifies a threshold deformation leading to lamina rupture and induction of specific genes. Integr Biol (Camb) 4, 1406–1414. 23038068 10.1039/c2ib20056b

[165] Yan‐Jun Liu A , Le Berre M , Voituriez R and Piel M (2015) Confinement and low adhesion induce fast amoeboid migration of slow mesenchymal cells. Cell 160, 659–672. 25679760 10.1016/j.cell.2015.01.007

[166] Elpers MA , Varlet AA , Agrawal R and Lammerding J (2023) Agarose‐based 3D cell confinement assay to study nuclear mechanobiology. Curr Protoc 3, e847. 37459474 10.1002/cpz1.847 PMC10407883

[167] Hanahan D (2022) Hallmarks of cancer: new dimensions. Cancer Discov 12, 31–46. 35022204 10.1158/2159-8290.CD-21-1059

[168] Nia HT , Munn LL and Jain RK (2020) Physical traits of cancer. Science 370, eaaz0868. 33122355 10.1126/science.aaz0868 PMC8274378

[169] Massey A , Stewart J , Smith C , Parvini C , McCormick M , Do K and Cartagena‐Rivera AX (2024) Mechanical properties of human tumour tissues and their implications for cancer development. Nat Rev Phys 64, 269–282. 10.1038/s42254-024-00707-2 PMC11066734 38706694

[170] Hyun J and Kim HW (2022) Leveraging cellular mechano‐responsiveness for cancer therapy. Trends Mol Med 28, 155–169. 34973934 10.1016/j.molmed.2021.11.006

[171] Damiano JS , Cress AE , Hazlehurst LA , Shtil AA and Dalton WS (1999) Cell adhesion mediated drug resistance (CAM‐DR): role of integrins and resistance to apoptosis in human myeloma cell lines. Blood 93, 1658–1667. 10029595 PMC5550098

[172] Shin JW and Mooney DJ (2016) Extracellular matrix stiffness causes systematic variations in proliferation and chemosensitivity in myeloid leukemias. Proc Natl Acad Sci USA 113, 12126–12131. 27790998 10.1073/pnas.1611338113 PMC5086998

[173] Lam WA , Rosenbluth MJ and Fletcher DA (2007) Chemotherapy exposure increases leukemia cell stiffness. Blood 109, 3505–3508. 17179225 10.1182/blood-2006-08-043570 PMC1852256

[174] Laperrousaz B , Berguiga L , Nicolini FE , Martinez‐Torres C , Arneodo A , Satta VM and Argoul F (2016) Revealing stiffening and brittling of chronic myelogenous leukemia hematopoietic primary cells through their temporal response to shear stress. Phys Biol 13, 03LT01. 10.1088/1478-3975/13/3/03LT01 27254599

[175] Ly C , Ogana H , Kim HN , Hurwitz S , Deeds EJ , Kim YM and Rowat AC (2023) Altered physical phenotypes of leukemia cells that survive chemotherapy treatment. Integr Biol (Camb) 15, zyad006. 37247849 10.1093/intbio/zyad006

[176] Yu W , Sharma S , Rao E , Rowat AC , Gimzewski JK , Han D and Rao J (2022) Cancer cell mechanobiology: a new frontier for cancer research. J Natl Cancer Cent 2, 10–17. 39035217 10.1016/j.jncc.2021.11.007 PMC11256617

[177] Paula Zambuzi Cardoso Marsola A , Pinto Simões B , Carvalho Palma L , Gabriela Berzoti‐Coelho M , Mara Burin S and Attié de Castro F (2018) Expression of Hippo signaling pathway and Aurora kinase genes in chronic myeloid leukemia. Med Oncol 35, 26. 29387948 10.1007/s12032-018-1079-6

[178] Li H , Huang Z , Gao M , Huang N , Luo Z , Shen H , Wang X , Wang T , Hu J and Feng W (2016) Inhibition of YAP suppresses CML cell proliferation and enhances efficacy of imatinib in vitro and in vivo. J Exp Clin Cancer Res 35, 1–11. 27599610 10.1186/s13046-016-0414-z PMC5012077

[179] Wu R , Yang H , Wan J , Deng X , Chen L , Hao S and Ma L (2018) Knockdown of the hippo transducer YAP reduces proliferation and promotes apoptosis in the Jurkat leukemia cell. Mol Med Rep 18, 5379–5388. 30320399 10.3892/mmr.2018.9556 PMC6236312

[180] Cottini F , Hideshima T , Xu C , Sattler M , Dori M , Agnelli L , Ten Hacken E , Bertilaccio MT , Antonini E , Neri A *et al*. (2014) Rescue of Hippo coactivator YAP1 triggers DNA damage–induced apoptosis in hematological cancers. Nat Med 20, 599–606. 24813251 10.1038/nm.3562 PMC4057660

[181] Zhou X , Chen N , Xu H , Zhou X , Wang J , Fang X , Zhang Y , Li Y , Yang J , Wang X *et al*. (2020) Regulation of hippo‐YAP signaling by insulin‐like growth factor‐1 receptor in the tumorigenesis of diffuse large B‐cell lymphoma. J Hematol Oncol 13, 77. 32546241 10.1186/s13045-020-00906-1 PMC7298789

[182] Zhang F , Issah MA , Fu HY , Zhou HR , Liu TB and Shen JZ (2024) LATS1 promotes B‐ALL tumorigenesis by regulating YAP1 phosphorylation and subcellular localization. Curr Med Sci 44, 81–92. 38277019 10.1007/s11596-023-2821-7

[183] Walling BL and Murphy PM (2021) Protean regulation of leukocyte function by nuclear Lamins. Trends Immunol 42, 323–335. 33653660 10.1016/j.it.2021.02.005

[184] Agrelo R , Setien F , Espada J , Artiga MJ , Rodriguez M , Pérez‐Rosado A , Sanchez‐Aguilera A , Fraga MF , Piris MA and Esteller M (2005) Inactivation of the Lamin A/C gene by CpG Island promoter hypermethylation in hematologic malignancies, and its association with poor survival in nodal diffuse large B‐cell lymphoma. J Clin Oncol 23, 3940–3947. 15867203 10.1200/JCO.2005.11.650

[185] Kim HN , Ruan Y , Ogana H and Kim Y‐M (2020) Cadherins, selectins, and integrins in CAM‐DR in leukemia. Front Oncol 10, 592733. 33425742 10.3389/fonc.2020.592733 PMC7793796

[186] Astier AL , Xu R , Svoboda M , Hinds E , Munoz O , De Beaumont R , Crean CD , Gabig T and Freedman AS (2003) Temporal gene expression profile of human precursor B leukemia cells induced by adhesion receptor: identification of pathways regulating B‐cell survival. Blood 101, 1118–1127. 12393420 10.1182/blood-2002-05-1519

[187] Lapieaux L , Delforfe A , Bron P , De Bruyn C and Slryckmans P (1997) Chronic lymphocytic leukemia (CLL) B‐lymphocytes but not normal B‐lymphocytes are rescued from apoptosis by contact with bone marrow stromal cells. Exp Hematol 25, 862.

[188] Sonoda Y , Matsumoto Y , Funakoshi M , Yamamoto D , Hanks SK and Kasahara T (2000) Anti‐apoptotic role of focal adhesion kinase (FAK): induction of inhibitor‐of‐apoptosis proteins and apoptosis suppression by the overexpression of FAK in a human leukemic cell line, HL‐60. J Biol Chem 275, 16309–16315. 10821872 10.1074/jbc.275.21.16309

[189] Sbrana FV , Fiordi B , Bordini J , Belloni D , Barbaglio F , Russo L , Scarfò L , Ghia P and Scielzo C (2023) PYK2 is overexpressed in chronic lymphocytic leukaemia: a potential new therapeutic target. J Cell Mol Med 27, 576–586. 36747338 10.1111/jcmm.17688 PMC9930416

[190] Wang‐Gillam A , Lim KH , McWilliams R , Suresh R , Lockhart AC , Brown A , Breden M , Belle JI , Herndon J , Bogner SJ *et al*. (2022) Defactinib, pembrolizumab, and gemcitabine in patients with advanced treatment refractory pancreatic cancer: a phase I dose escalation and expansion study. Clin Cancer Res 28, 5254–5262. 36228156 10.1158/1078-0432.CCR-22-0308 PMC9772237

[191] Wigton EJ , Thompson SB , Long RA and Jacobelli J (2016) Myosin‐IIA regulates leukemia engraftment and brain infiltration in a mouse model of acute lymphoblastic leukemia. J Leukoc Biol 100, 143–153. 26792819 10.1189/jlb.1A0815-342R PMC5627497

[192] Jbireal JMA , Strell C , Niggemann B , Zänker K and Entschladen F (2010) The selective role of myosin VI in lymphoid leukemia cell migration. Leuk Res 34, 1656–1662. 20493527 10.1016/j.leukres.2010.04.018

[193] Estrada‐Abreo LA , Rodríguez‐Cruz L , Garfias‐Gómez Y , Araujo‐Cardenas JE , Antonio‐Andrés G , Salgado‐Aguayo AR , Orozco‐Ruiz D , Torres‐Nava JR , Díaz‐Valencia JD , Huerta‐Yépez S *et al*. (2021) High expression of myosin 1g in pediatric acute lymphoblastic leukemia. Oncotarget 12, 1937–1945. 34548909 10.18632/oncotarget.28055 PMC8448507

[194] Rousselot P , Mollica L , Guilhot J , Guerci A , Nicolini FE , Etienne G , Legros L , Charbonnier A , Coiteux V , Dartigeas C *et al*. (2021) Dasatinib dose optimisation based on therapeutic drug monitoring reduces pleural effusion rates in chronic myeloid leukaemia patients. Br J Haematol 194, 393–402. 34195988 10.1111/bjh.17654

[195] Gerber DE , Camidge DR , Morgensztern D , Cetnar J , Kelly RJ , Ramalingam SS , Spigel DR , Jeong W , Scaglioni PP , Zhang S *et al*. (2020) Phase 2 study of the focal adhesion kinase inhibitor defactinib (VS‐6063) in previously treated advanced KRAS mutant non‐small cell lung cancer. Lung Cancer 139, 60–67. 31739184 10.1016/j.lungcan.2019.10.033 PMC6942685

[196] Nicolas‐Boluda A , Vaquero J , Vimeux L , Guilbert T , Barrin S , Kantari‐Mimoun C , Ponzo M , Renault G , Deptula P , Pogoda K *et al*. (2021) Tumor stiffening reversion through collagen crosslinking inhibition improves T cell migration and anti‐PD‐1 treatment. elife 10, 1–29. 10.7554/eLife.58688 PMC8203293 34106045

[197] Jiang Y , Zhang H , Wang J , Liu Y , Luo T and Hua H (2022) Targeting extracellular matrix stiffness and mechanotransducers to improve cancer therapy. J Hematol Oncol 15, 1–15. 35331296 10.1186/s13045-022-01252-0 PMC8943941

[198] Zuela‐Sopilniak N and Lammerding J (2022) Can't handle the stress? Mechanobiology and Disease. Trends Mol Med 28, 710. 35717527 10.1016/j.molmed.2022.05.010 PMC9420767

